# Repeated Exposure to Sevoflurane in Neonatal Mice Induces Cognitive and Synaptic Impairments in a TTLL6‐Mediated Tubulin Polyglutamylation Manner

**DOI:** 10.1111/cns.70376

**Published:** 2025-04-09

**Authors:** Yang Yu, Yue Zhao, Jingyu Feng, Naqi Lian, Jiafeng Yu, Yongyan Yang, Junyan Yao, Yonghao Yu

**Affiliations:** ^1^ Department of Anaesthesia Tianjin Medical University General Hospital Tianjin China; ^2^ Tianjin Institute of Anaesthesiology Tianjin China; ^3^ Department of Anaesthesiology Shanghai East Hospital, School of Medicine, Tongji University Shanghai China

**Keywords:** cognitive impairment, neonatal mice, sevoflurane, synaptic plasticity, tubulin polyglutamylation

## Abstract

**Aims:**

Repeated sevoflurane exposure during the neonatal stage may induce Tau phosphorylation, dendritic spine loss, and neurocognitive impairment in the developing brain. Tubulin tyrosine ligase like‐6 (TTLL6), which aggregates in dendrites due to Tau missorting, regulates microtubule stability via α‐tubulin polyglutamylation. Meanwhile, Spastin modulates dendritic spine formation by severing microtubules. We hypothesize that repeated sevoflurane treatment impairs dendritic spine remodeling in neonatal mice by enhancing TTLL6‐mediated tubulin polyglutamylation and increasing Spastin expression, leading to cognitive dysfunction in their pre‐adolescent stage.

**Methods:**

Six‐day‐old wild type (WT), TTLL6 brain conditional knockout (TTLL6_CKO_), TTLL6‐flox (TTLL6_CON_) and Tau‐knockout mice were treated with 3% sevoflurane for 2 h daily on postnatal days (P) 6, 8, and 10. Levels of Tau, phosphorylated Tau (pTau), TTLL6, polyglutamylated tubulin, ATP, Spastin, PSD95, Tau‐TTLL6 interaction, Tau‐TTLL6 missorting, dendritic spine remodeling, and behavioral alterations were compared across these groups.

**Results:**

Repeated sevoflurane exposure during brain development in neonatal mice could reduce dendritic spine density, synapse number, PSD95, and ATP levels, while increasing pTau, polyglutamylated tubulin, Tau‐TTLL6 missorting from axons to the somatodendritic compartment, and Spastin levels, leading to cognitive impairment later in their pre‐adolescent stage (P30). However, these changes were ameliorated in the TTLL6_CKO_ mice.

**Conclusions:**

Repeated neonatal sevoflurane exposure results in synaptic impairment through TTLL6‐mediated tubulin polyglutamylation and increased Spastin expression, causing pre‐adolescent cognitive dysfunction in mice. This process is initiated by Tau phosphorylation and missorting from axons to somatodendritic compartments.

## Graphical abstract

Our findings suggest that sevoflurane treatment in neonatal mice may cause cognitive impairment by altering dendritic spine density and maturation in the CA3 region of the hippocampus. This effect is mediated by TTLL6‐induced tubulin polyglutamylation and Spastin overexpression, driven by Tau phosphorylation and missorting from axons to the somatodendritic compartment.

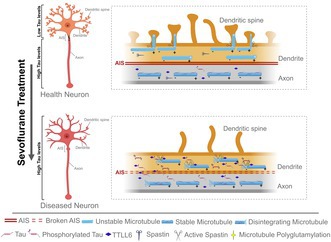

**Abbreviations**
ad
: Alzheimer's disease
CO‐IP: co‐immunoprecipitation
GAPDH: glyceraldehyde 3‐phosphate dehydrogenase
IB: immunoblotting
IP: immunoprecipitation
MBT: Microtubule‐bound Tau
MTS: microtubules
MUT: microtubule‐unbound Tau
MWM: Morris water maze
P: postnatal
PSD: postsynaptic density
PSD95: postsynaptic density protein‐95
pTau: Phosphorylated Tau
RT‐PCR: reverse transcriptase polymerase chain reaction
SP: stratum pyramidale
Spastin‐PS101: Phosphorylation of Spastin at serine 101
Spastin‐PS595: Phosphorylation of Spastin at serine 595
Spastin‐PS99: Phosphorylation of Spastin at serine 99
Spastin‐PT598: Phosphorylation of Spastin at threonine 598
SR: stratum radiatum area
TTLL6: tubulin tyrosine ligase like‐6
TTLL6_CKO_
: TTLL6 brain tissue conditional knockout mice
TTLL6_CON_
: TTLL6‐flox mice
WT: wild type

## 1 Introduction

The increasing use of general anesthesia in infants for medical procedures has raised concerns about the safety of young children, posing a significant public health issue owing to advancements in technology [1, 2]. Sevoflurane is the most frequently used inhalational anesthetic [3]. However, repeated or long‐term exposure to this medication before the age of 3–4 years may lead to a higher risk of long‐term adverse reactions such as behavioral, learning, and cognitive impairment [4, 5, 6], although contradictory data exist [7, 8].

The nervous system in rodents develops rapidly from the last 2 days of pregnancy to approximately 2 weeks after delivery, with a peak at 6–7 days after birth [9, 10]. During this period, the brain mass increases quickly, axons lengthen swiftly, dendrites sprout rapidly, and numerous synapses develop [9]. Exposure of the growing brain to general anesthetics during this period may cause neuronal death and dendritic spine formation disorder, disrupting neural development and synapse formation, and resulting in long‐term brain impairment [10, 11]. Recent research indicates that the flexibility of dendritic spines in the hippocampus plays an important role in repeated sevoflurane exposure‐induced brain neurodevelopmental damage in young animals [12, 13, 14]. Moreover, our previous research showed that 3% sevoflurane anesthesia, administered 2 h a day for 3 days, induces Tau phosphorylation, dendritic spine loss, and neurocognitive impairment in neonatal [postnatal (P) 6] but not in adult (P60) mice [3, 15, 16, 17, 18]. However, the mechanisms underlying these age‐dependent effects remain unclear.

Microtubules (MTS) are polarized cytoskeletal filaments important for neuronal development and function. They are related to neuron polarity, axon growth, and dendritic stability, and act as tracks for the intracellular transport of neurones. Moreover, dendritic spine activity is dependent on microtubule invasion, which promotes dendritic spine maturation and enlargement of the dendritic spine head (forming mushroom‐shaped and short, thick spines) [19, 20]. Tau is an axonal microtubule‐related protein that promotes Tubulin polymerization, stabilizes microtubule structure, and maintains neuronal function [21]. In normal mature neurones, Tau binds to microtubules and is mainly stored in axons. However, in diseased or stressed neurones, Tau is phosphorylated and separated from microtubules and then mis‐sorted into the soma and dendrites [22]. Our previous research showed that neonatal mice express higher levels of microtubule‐unbound Tau (MUT) [15], and some evidence suggests that MUT may be the main cause of abnormal phosphorylation of Tau [22, 23, 24]. These observations indicate that newborn mice are more vulnerable to sevoflurane‐induced neurotoxicity. Tubulin tyrosine ligase like‐6 (TTLL6), a member of the TTLL family, is primarily found in neuronal axons and regulates microtubule stability by modulating polyglutamylation [25]. Research on primary neuronal models of Alzheimer's disease (AD) indicates that Tau and TTLL6 proteins interact structurally, with Tau mislocalization leading to TTLL6 protein aggregation in the dendritic compartment [26]. Additionally, Spastin is a member of the AAA ATP enzyme family that regulates dendritic spine formation by severing microtubules and is highly conserved [27, 28]. In the presence of ATP, the AAA domain of Spastin forms a hexamer that binds to the negatively charged C‐terminus of microtubule proteins and causes microtubule severing by pulling the minus end of the microtubule. Members of the TTLL family can modulate Spastin expression via tubulin polyglutamylation [26, 27].

In this study, we aimed to investigate the impairment of dendritic spine plasticity as a mechanism of developmental brain neurotoxicity induced by repeated sevoflurane anesthesia. Our hypothesis suggests that the impairments observed in this case were caused by a pathological sequence of events, where Tau, associated with microtubules in the axons, is abnormally located in the somatodendritic region of neurons. Consequently, this leads to the dendritic mislocalization of TTLL6, polyglutamylation of tubulin, and finally, the activation of more Spastin, resulting in microtubule severing and synaptic plasticity impairments.

## 2 Materials and Methods

### 2.1 Mice, Anesthesia, and Treatment

The Animal Experimental Ethics Committee of Tianjin Medical University General Hospital in Tianjin, China approved the animal protocol (Approval No. IRB2020‐DW‐36). Minimizing the use of animals in scientific research is imperative. Adult wild type C57BL/6J mice (WT) of 2monthsold were obtained from Sipeifu Bioscience (license number, SCXK 2019‐0010; Beijing, China). Adult TTLL6 floxed mice (TTLL6_CON_: TTLL6^f/f^), Camk2‐Cre mice, and Tau knockout mice (Tau‐KO) were acquired from the Shanghai Model Organisms Centre Inc. (license number, SCXK 2019‐0002; Shanghai, China). Male heterozygous Camk2‐Cre mice were crossed with female TTLL6^f/f^ mice to obtain TTLL6 conditional knockout mice (TTLL6_CKO_: Camk2‐Cre^+^; TTLL6^f/f^) with specific deletions in the hippocampal and cortical cells. Neonatal mice at postnatal day 6 were obtained from our own breeding, and only female mice were used in the research. The genotype of the transgenic mice was determined by analyzing genomic DNA obtained from their tails.

A total of 102 wild type, 102 TTLL6_CON_, 102 TTLL6_CKO_, and 18 Tau‐KO neonatal mice were utilized in the study. Neonatal mice were induced with a mixture of 60% O_2_ and 3% sevoflurane for 2 h. The gas concentrations in the chambers were maintained at the set values, and the mice rectal temperature was maintained at 37°C ± 0.5°C by controlling the temperature. The sevoflurane group received 3% sevoflurane and 60% O_2_ for 2 h/day on P6, P8, and P10, whereas the control group received only 60% O_2_ for 2 h/day on P6, P8, and P10, as described by Lu et al. [10]. The experiments were conducted simultaneously on each day. After the modeling was completed, the mice were returned to their mother mouse's cage once they exhibited stable breathing and were fully awake. Notably, blood gas values are not significantly affected by the use of 3% sevoflurane and 60% O_2_ during anesthesia [15].

### 2.2 Primary Neurons Culture and Treatment

Mouse embryos at 15 days of gestation were decapitated and euthanized in various experimental groups. Mouse hippocampal primary neurons were cultured as described previously [3]. Please see the Supporting Information.

### 2.3 Open‐Field Test

The open‐field test was performed as described previously [29]. Please see the Supporting Information.

### 2.4 Morris Water Maze

The Morris water maze (MWM) test was conducted at P30 (pre‐adolescent stage) for 7 days (P30‐36) with four trials each day. Details of the Morris water maze test have been described previously [15]. Please see the Supporting Information.

### 2.5 Brain Tissue Harvest, Lysis, and Protein Quantification

The mice were decapitated after P10 sevoflurane anesthesia, and their hippocampi were harvested. Please see the Supporting Information.

### 2.6 Reverse Transcriptase Polymerase Chain Reaction (RT‐PCR)

Spastin mRNA levels were measured and normalized to glyceraldehyde 3‐phosphate dehydrogenase (GAPDH). Mouse Spastin primers (ID No. QT01040893) and mouse GAPDH primers (ID No. QT01658692). Please see the Supporting Information.

### 2.7 Microtubule Binding Assay

A microtubule‐binding protein spin‐down assay kit (Cat# BK029; Cytoskeleton, Denver, CO, USA) was used. Please see the Supporting Information.

### 2.8 Western Blot

Total Tau and TTLL6 expression levels were detected using anti‐Tau5 antibody (Cat# ab80579, 55 kDa, 1:1000, Abcam) and anti‐TTLL6 antibody (Cat# DF12178, 55 kDa, 1:1000, Affinity Biosciences), respectively. Spastin antibody (Cat# ab244354, 67 kDa, 1:1000; Abcam) was used to measure Spastin protein expression levels. The AT8 antibody (Tau‐PS202/PT205, Cat# MN1020, 55 kDa, 1:2000, Thermo Fisher Scientific) was used to detect Tau phosphorylated at its serine 202 and threonine 205 residues. The PSD95 antibody (Cat# ab13552, 100 kDa, 1:1000, Abcam) was used to measure PSD95 protein expression levels. α‐Tubulin (polyglutamylated) antibody (Cat# T9822, 50 kDa, 1:1000, Sigma‐Aldrich) was used to measure polyglutamylated Tubulin expression levels. Finally, an antibody specific for the protein GAPDH (Cat# AF7021, 37 kDa, 1:5000, Sigma, Affinity Biosciences) was utilized as a reference to determine variations in the total protein quantity during loading. The quantification of the Western blot was performed using the method described previously [30]. Please see the Supporting Information.

### 2.9 Co‐Immunoprecipitation

Co‐immunoprecipitation (CO‐IP) experiments were performed using mouse hippocampal tissue proteins to investigate the interaction between Tau and TTLL6. Please see the Supporting Information.

### 2.10 Golgi Staining and Dendritic Spine Density Analysis

Golgi staining was performed on P10 mice. Staining and analysis were performed using a Golgi staining reagent kit (Cat#: PK401, FD Neuro Technologies, USA), as described in our earlier study [30].

### 2.11 Electron Microscope

The brain sections from 10‐day‐old mice were then stained with uranyl acetate and lead citrate, air‐dried overnight, and examined under an electron microscope. Please see the Supporting Information.

### 2.12 Immunohistochemistry

Phosphorylated Tau antibody (AT8, Cat# MN1020, 55 kDa, 1:2000, Thermo Fisher Scientific), TTLL6 antibody (Cat# DF12178, 1:100, Affinity Biosciences), Spastin antibody (Cat# ab244354, 1:100, Abcam), and α‐Tubulin antibody (Cat# ab6160, 1:50, Abcam) were used to measure expression levels and distribution of these four proteins in the CA3 region of the hippocampal tissue and primary neurons. Immunofluorescence experiments were performed as described previously [3, 31]. Please see the Supporting Information.

### 2.13 Multiplexed Quantitative Mass Spectrometry‐Based Phosphoproteomics

Whole cerebral tissues were collected following the cessation of repeated sevoflurane anesthesia. Multiplexed phosphoproteomic analysis utilizing mass spectrometry was performed in accordance with established protocols [15]. For detailed methodology, please refer to the Supporting Information.

### 2.14 ATP Measurement

The levels of ATP in the hippocampal and cortical tissues of mice were measured using the ATP Colorimetric/Fluorometric Assay Kit and the methods described in our previous studies [15]. Please see the Supporting Information.

### 2.15 Statistics

Data analyses were performed using GraphPad Prism (version 9.0) and SPSS statistical software (version 21.0). Data obtained from biochemistry studies, centre time of open field test, total distance of open‐field test, and escape latency of MWM test is presented as mean ± standard deviation (SD). The numbers of platform crossings in the MWM are presented as medians with interquartile ranges. The number of mice was 10 in each group for behavioral studies, 6 in each group for western blotting, PCR, and ATP measurements, 4 in each group for mass spectrometry studies, and 3 in each group for the microtubule‐binding assay, co‐immunoprecipitation, Golgi staining, electron microscopy, and immunohistochemistry. These numbers were selected based on the results of our previous studies [15, 16]. The interaction between time and group factors was determined using a two‐way ANOVA with repeated measurements to analyze the difference in learning curves (based on escape latency) between mice in the control group and those treated with anesthesia in the MWM. A post hoc Bonferroni test was used to compare the difference in escape latency between the control and anesthesia groups on each day of the MWM. The Mann–Whitney *U* test was used to determine the difference in platform crossing times between sevoflurane anesthesia and control conditions. There were no missing data for the variables of the open‐field test (centre time and total distance) and MWM (escape latency and platform crossing times) during the data analysis. Finally, to compare the two groups for other biochemical data, the unpaired *t*‐test (if the values were in a Gaussian distribution) or the Mann–Whitney test (non‐Gaussian distribution) was applied. The normality of each group of data was assessed using the Shapiro–Wilk test. Statistical significance was expressed as *p* < 0.05.

## 3 Results

### 3.1 Repeated Sevoflurane Exposure in Neonatal Mice Induced Cognitive and Synaptic Impairments in the Developing Brain

**FIGURE 1:**
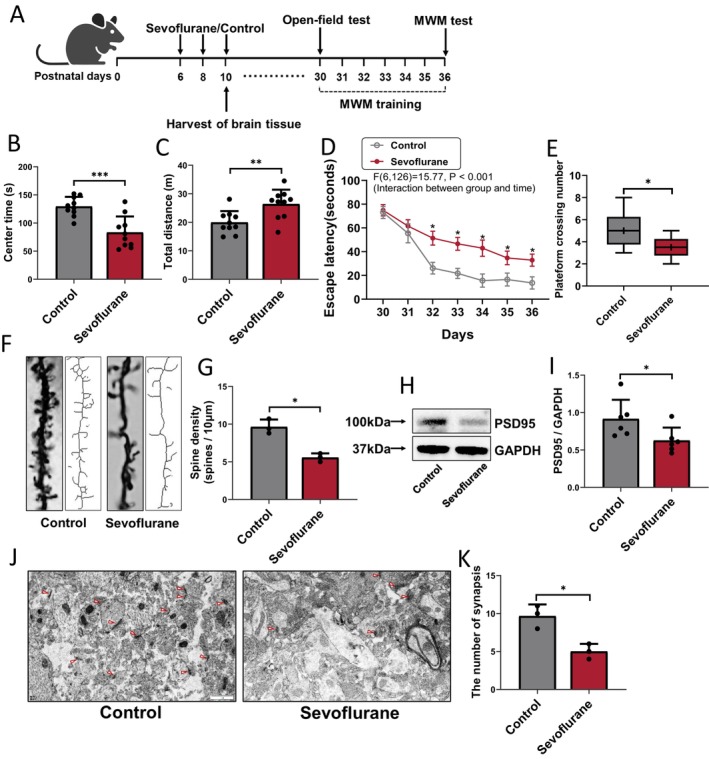
Effects of repeated sevoflurane exposure in neonatal mice on cognitive and synaptic plasticity functions in developing brain. (A) Experimental design: Neonatal mice (P6) were administered 3% sevoflurane +60% O_2_ or 60% O_2_ alone for 2 h every day for 3 days (P6, P8, and P10). Hippocampus tissues were collected on P10 at the conclusion of the sevoflurane or control condition. Behavioral tests were conducted from P30 to P36 (open‐field test: P30; MWM test: P36; MWM training: P30–P36). Summary of (B) centre time and (C) total distance in the open‐field test after control or repetitive sevoflurane treatment; *n* = 10 mice/group. Navigation test and probe trial were respectively used to compare the (D) escape latency (P30–P36) and (E) platform crossing number (P36) between the two groups (control and sevoflurane); *n* = 10 mice/group. (F) Representative original photomicrographs and skeletonized dendrites images, and (G) qualification of Golgi‐stained dendrites in the CA3 region, *n* = 3 mice/group. (H) PSD95 protein expression and (I) qualification in the hippocampus after control or repetitive sevoflurane treatment; *n* = 6 mice/group. (J) Electron microscope showing the number of synapses (red triangles mark the postsynaptic density (PSD), serving as a marker for synapse counting), scale bar: 1 μm. (K) Qualification of synapsis numbers, *n* = 3 mice/group. *p < 0.05, **p < 0.01, ***p < 0.001.

We examined the cognitive function and synaptic plasticity of mice after repeated sevoflurane anesthesia or control conditions on P6, 8, and 10, as described in Figure 1A. During a 10‐min open‐field test, repeated sevoflurane‐treated mice spent less time in the center of an open field (Treated vs. Control; 83.53 ± 28.54 s vs. 129.90 ± 17.06 s; *p* < 0.001) (Figure 1B) and traveled substantially farther than control mice (Treated vs. Control; 26.39 ± 5.05 m vs. 19.96 ± 3.97 m; *p* = 0.005) (Figure 1C), indicating that repeated sevoflurane treatment elicited anxiety‐like behavior. In the MWM test, mice exposed to multiple sevoflurane treatments had higher escape latency in days 32–36 (Treated vs. Control; *F* = 15.77, *p* < 0.001, interaction between group and time) (Figure 1D) and lower platform crossing times (Treated vs. Control; 3.50 [1.50] vs. 5.00 [2.50]; *p* = 0.046) (Figure 1E) than those of mice in control conditions, indicating that repeated neonatal sevoflurane exposure induced spatial learning and memory impairment in their pre‐adolescent stage.

Golgi staining of the cerebral hippocampus in the CA3 region was detected 10 days after treatment (Figure 1F). Quantitative analysis revealed that multiple inhalation treatments with sevoflurane significantly reduced the density of dendritic spines in the CA3 area of the hippocampus in P10 mice (Treated vs. Control; 5.57 ± 0.55 vs. 9.63 ± 0.97; *p* = 0.023) (Figure 1G). In addition, western blotting showed that PSD95 protein expression levels were lower in the sevoflurane group than in the control group (Treated vs. Control; 0.63 ± 0.17 vs. 0.91 ± 0.26; *p* = 0.04) (Figure 1H,I). Moreover, the number of synapses in the hippocampal CA3 area of P10 mice was evaluated using electron microscopy (Figure 1J). The number of synapses in neonatal mice decreased considerably following repeated sevoflurane anesthesia (Treated vs. control; 5.00 ± 1.00 vs. 9.67 ± 1.53; *p* = 0.023) (Figure 1K).

These findings indicate that repeated neonatal sevoflurane anesthesia leads to cognitive impairment in pre‐adolescent mice by modulating the density and maturity of dendritic spines in the CA3 region of the hippocampus.

### 3.2 Repeated Sevoflurane Exposure Increased Spastin Expression in Hippocampal Neurons of Neonatal Mice

**FIGURE 2:**
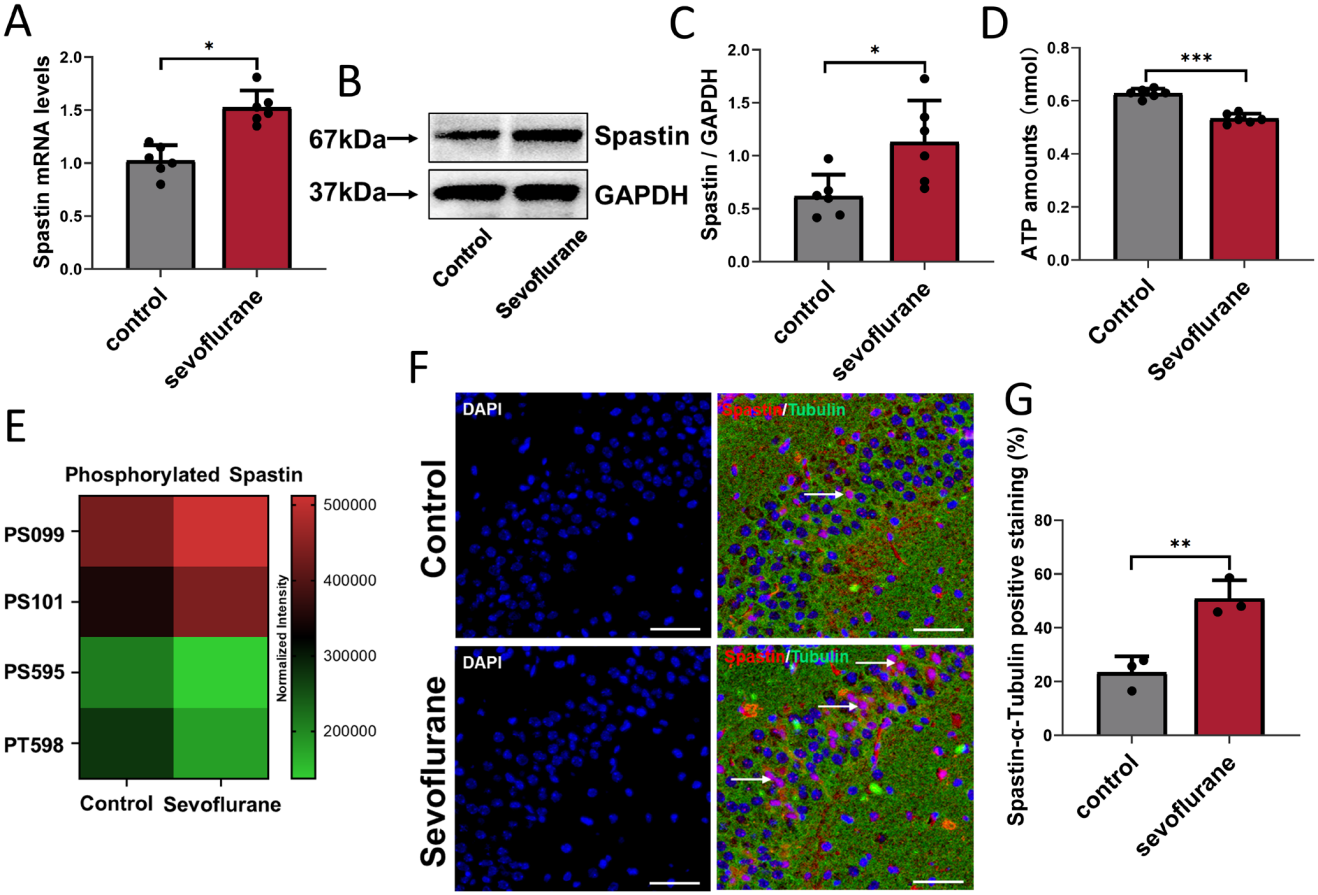
Effects of repeated sevoflurane exposure on Spastin expression in the hippocampus of neonatal mice. (A) Spastin mRNA and (B) Spastin protein expression and (C) qualification in the hippocampus after control or repetitive sevoflurane treatment; *n* = 6 mice/group. (D) ATP expression levels in the hippocampus of neonatal mice in Control and Sevoflurane groups; *n* = 3 mice/group. (E) Phosphorylated sites of Spastin expression after control or repetitive sevoflurane treatment; *n* = 6 mice/group. (F) Immunofluorescence of the protein labeled by Spastin (red) and α‐tubulin (green) in the CA3 area of the hippocampus after control or repetitive sevoflurane treatment, Spastin‐α‐tubulin positive staining (pink) was marked with white arrows, scale bar: 50 μm, blue is DAPI. (G) Qualification of Spastin‐α‐tubulin positive staining (%) in different groups; *n* = 3 biological replicates. *p < 0.05, **p < 0.01, ***p < 0.001.

Spastin, a member of the AAA ATPase family, regulates dendritic spine formation by severing the microtubules [27, 28]. Therefore, we evaluated Spastin protein expression levels in brain tissues and primary neurones (Figure S1A), given either a control or repeated sevoflurane treatment. Sevoflurane increased Spastin mRNA (Treated vs. Control; 1.53 ± 0.16 vs. 1.03 ± 0.14; *p* < 0.001) (Figure 2A) and protein (Treated vs. Control; 1.13 ± 0.39 vs. 0.62 ± 0.20; *p* = 0.02) (Figure 2B,C) expression levels in P10 mice. Furthermore, ATP expression levels were lower in the sevoflurane group than in the control group of P10 mice (Treated vs. control; 0.53 ± 0.02 vs. 0.63 ± 0.02; *p* < 0.001) (Figure 2D), indicating that excessive activation of Spastin may be related to the consumption of ATP. Moreover, sevoflurane increased phosphorylated Spastin expression levels at serine 99 (PS99) and serine 101 (PS101); however, it decreased phosphorylated Spastin expression levels at serine 595 (PS595) and threonine 598 (PT598) (Figure 2E) in the brain tissues of P10 mice. In addition, immunostaining of primary neurones showed that sevoflurane treatment could increase Spastin expression both in the cell body and dendrites, and the increase in Spastin may break down the α‐tubulin in dendrites (Figure S1B). Moreover, immunostaining of the hippocampus revealed that neonatal mice that were administered repeated sevoflurane treatment had more Spastin‐α‐tubulin positive staining in the CA3 area of the hippocampus than those in control mice (Treated vs. Control; 50.85% ± 6.83% vs. 23.36% ± 6.00%; *p* = 0.024) (Figure 2F,G).

Taken together, repetitive sevoflurane administration increased Spastin levels and activation in dendrites, which might explain microtube reduction in the brains of neonatal mice. The activation of Spastin consumes large quantities of ATP and could be originated by a greater expression of Spastin‐PS99 and PS101 or a lesser degree of Spastin‐PS595 and PT598.

### 3.3 Repeated Sevoflurane Exposure Enhanced Tau and TTLL6 Missorting in Hippocampal Neurons of Neonatal Mice

**FIGURE 3:**
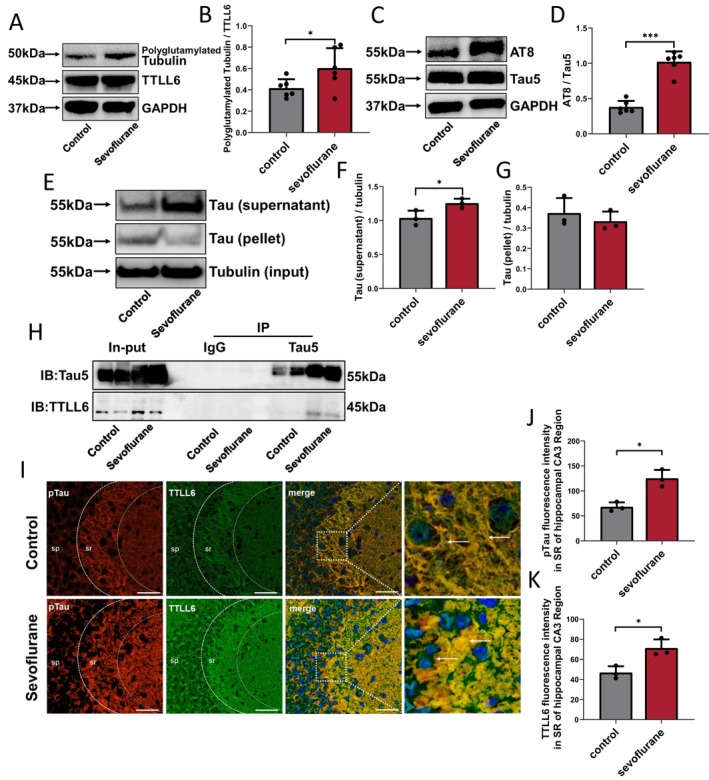
Effects of repeated sevoflurane exposure on Tau and TTLL6 missorting in hippocampus of neonatal mice. (A) Expression of polyglutamylated Tubulin and TTLL6 in the hippocampus after control or repetitive sevoflurane treatment. (B) Qualification of polyglutamylated tubulin/TTLL6 in different groups; *n* = 6 mice/group. (C) Expression of AT8 and Tau5 in the hippocampus after control or repetitive sevoflurane treatment. (D) Qualification of AT8/Tau5 in different groups; *n* = 6 mice/group. (E) Results of microtubule binding assay in the hippocampus of neonatal mice in Control and Sevoflurane groups, (F) the supernatanted protein representing microtubule‐unbound Tau, (G) the pelleted protein representing microtubule‐bond Tau; *n* = 3 mice/group. (H) Immunoprecipitation analysis of Tau5 and TTLL6 interaction in hippocampus cells. IgG: Negative control; Input: Total protein and positive control; IB: Immunoblotting; IP: Immunoprecipitation; *n* = 3 biological replicates. (I) Immunofluorescence of the protein labeled by pTau (red) and TTLL6 (green) in the CA3 area of hippocampus after control or repetitive sevoflurane treatment, repeated sevoflurane treatment increased pTau‐TTLL6 co‐expressed levels (yellow) in cell body and dendrites (marked with write arrows); blue is DAPI, scale bar: 50 μm. Qualification of (J) pTau and (K) TTLL6 fluorescence intensity in SR area of CA3 region in different groups; *n* = 3 biological replicates. SR, Stratum radiatum area: Composed mostly of apical dendrites of pyramidal neurons; SP: Stratum pyramidale: Composed mostly of tightly arranged pyramidal cells. *p < 0.05, **p < 0.01, ***p < 0.001.

Next, we examined the mechanisms by which repeated sevoflurane administration increases Spastin‐induced microtubule disruption. Zempel et al. reported that Spastin can be recruited to microtubules by polyglutamylation of α‐tubulin conferred by the TTLL family (TTLL6 and TTLL11) [26]. In our study, polyglutamylated α‐tubulin expression was higher in repetitive sevoflurane‐treated mice than in control‐exposed mice; however, no significant difference in TTLL6 levels was found (Figure 3A). The ratio of polyglutamylated Tubulin to TTLL6 was higher in the sevoflurane group than in the control group (Treated vs. Control; 0.62 ± 0.18 vs. 0.42 ± 0.08; *p* = 0.04) (Figure 3B). Furthermore, phosphorylated Tau (AT8) expression levels in the hippocampal tissues of mice were higher in the sevoflurane group than in the control group (AT8/Tau5; Treated vs. Control; 1.02 ± 0.15 vs. 0.38 ± 0.09; *p* < 0.001) (Figure 3C,D). The microtubule binding assay showed that there was more microtubule‐unbound Tau (supernatant, MUT) in the hippocampus of repetitive sevoflurane‐treated mice than in that of control mice (Treated vs. Control; 1.25 ± 0.07 vs. 1.04 ± 0.11; *p* = 0.023) (Figure 3E,F). There was no significant difference in microtubule‐bound Tau (pellet, MBT) expression between sevoflurane and control mice (Treated vs. Control; 0.33 ± 0.04 vs. 0.37 ± 0.04; *p* = 0.4) (Figure 3G). Co‐immunoprecipitation results validated the association between Tau and TTLL6 proteins, and repeated sevoflurane administration significantly promoted Tau‐TTLL6 binding (Figure 3H). Moreover, immunostaining of primary neurones revealed that Tau and TTLL6 were expressed mainly in axons under normal conditions; however, following repeated sevoflurane treatment, the levels of these two proteins increased and they migrated from axons to cell bodies and dendrites. Furthermore, the neurones in the sevoflurane group showed more dendritic branching than those in the control group (Figure S1C). Additionally, hippocampal immunostaining showed that pTau was mainly expressed in the cytoplasm, while TTLL6 was expressed in both the cytoplasm and nucleus; repeated sevoflurane treatment could increase the pTau‐TTLL6 fluorescence expression in the apical dendrites of pyramidal neurons of the hippocampal CA3 region (Figure 3I). Repeated sevoflurane exposure enhanced pTau (Treated vs. Control; 124.9 ± 17.12 vs. 67.79 ± 9.32; *p* = 0.02) (Figure 3J) and TTLL6 (Treated vs. Control; 71.07 ± 8.82 vs. 46.71 ± 6.42; *p* = 0.023) (Figure 3K) expression in the stratum radiatum area (SR: composed mostly of apical dendrites of pyramidal neurons) of the hippocampal CA3 region.

Altogether, repeated sevoflurane treatment increases MUT levels in hippocampal neurons, leading to the mislocalization of TTLL6 from the soma to the dendrites via a Tau‐dependent mechanism. This enhanced dendritic TTLL6 may catalyze the polyglutamylation of α‐tubulin by adding polyGlu residues to its C‐terminal tail.

### 3.4 TTLL6 Conditional Knock‐Out Reversed the Overexpression of Polyglutamylated Tubulin, but Not pTau Missorting, in the Hippocampus of Repetitive‐Anesthetized Neonatal Mice

**FIGURE 4:**
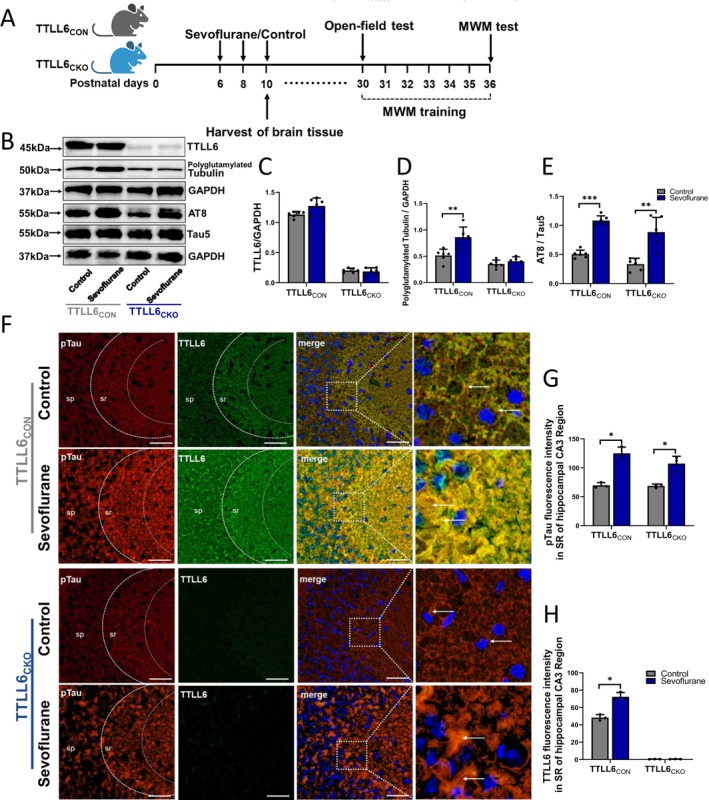
Effects of repeated sevoflurane exposure on related protein expression of Tau‐TTLL6 missorting in TTLL6_CON_ or TTLL6_CKO_ neonatal mice. (A) Experimental design: TTLL6_CON_ (TTLL6^f/f^) and TTLL6_CKO_ (Camk2‐Cre^+^; TTLL6^f/f^) mice were administered 3% sevoflurane + 60% O_2_ or 60% O_2_ alone for 2 h every day for 3 days (P6, P8, and P10). Hippocampus tissues were collected at the conclusion of the repetitive sevoflurane inhalation or control condition. Behavioral tests were conducted from P30 to P36 (open‐field test: P30; MWM test: P36; MWM training: P30–P36). (B) Expression of TTLL6, polyglutamylated Tubulin, AT8, and Tau5 in the hippocampus after control or repetitive sevoflurane treatment. Qualification of (C) TTLL6, (D) polyglutamylated Tubulin, and (E) AT8/Tau5 in different groups; *n* = 6 mice/group. (F) Immunofluorescence of the protein labeled by pTau (red) and TTLL6 (green) in the CA3 area of hippocampus after control or repetitive sevoflurane treatment. pTau‐TTLL6 co‐expressed levels (yellow) was marked with white arrows; blue is DAPI, scale bar: 50 μm. Qualification of (G) pTau and (H) TTLL6 fluorescence intensity in the SR area of the CA3 region in different groups; *n* = 3 biological replicates. SR, Stratum radiatum area: Composed mostly of apical dendrites of pyramidal neurons; SP, Stratum pyramidale: Composed mostly of tightly arranged pyramidal cells. *p < 0.05, **p < 0.01, ***p < 0.001.

To determine the molecular mechanism, TTLL6 conditional knockout mice with specific deletions in hippocampal and cortex cells (TTLL6_CKO_: Camk2‐Cre^+^; TTLL6^f/f^) and TTLL6 floxed mice (TTLL6_CON_: TTLL6^f/f^) were used in this study (Figure 4A). Western blotting showed no TTLL6 expression in the hippocampi of TTLL6_CKO_ mice (Figure 4B,C). After sevoflurane treatment, polyglutamylated Tubulin expression levels increased in the hippocampus of TTLL6_CON_ mice but not in TTLL6_CKO_ mice (Treated vs. Control in TTLL6_CON_ mice; 0.86 ± 0.19 vs. 0.52 ± 0.12; *p* = 0.004; Treated vs. Control in TTLL6_CKO_ mice; 0.40 ± 0.09 vs. 0.35 ± 0.08; *p* = 0.31) (Figure 4D). Moreover, in both TTLL6_CON_ and TTLL6_CKO_ mice, phosphorylated Tau (AT8) expression levels were higher in the sevoflurane group than in control groups (AT8/Tau5; Treated vs. Control in TTLL6_CON_ mice; 1.08 ± 0.08 vs. 0.51 ± 0.07; *p* < 0.001; Treated vs. Control in TTLL6_CKO_ mice; 0.88 ± 0.25 vs. 0.33 ± 0.10; *p* = 0.006) (Figure 4E). Furthermore, hippocampal immunostaining (Figure 4F) revealed that repeated sevoflurane treatment enhanced pTau fluorescence in the SR area of the CA3 region in both TTLL6_CON_ and TTLL6_CKO_ mice compared to the control group (Treated vs. Control in TTLL6_CON_ mice; 125.0 ± 10.82 vs. 69.67 ± 4.72; *p* = 0.02; Treated vs. Control in TTLL6_CKO_ mice; 107.3 ± 12.66 vs. 68.67 ± 3.21; *p* = 0.02) (Figure 4G). Notably, under repetitive sevoflurane treatment, TTLL6 expression was elevated exclusively in the SR region of TTLL6_CON_ mice (Treated vs. Control in TTLL6_CON_ mice; 72.33 ± 4.93 vs. 48.33 ± 3.51; *p* = 0.02), whereas no TTLL6 expression was observed in the hippocampus of TTLL6_CKO_ mice, neither in the control nor in the sevoflurane groups (Figure 4H).

To further test the relationship of TTLL6 and Tau, wild‐type (WT) and Tau‐KO mice (Tau‐KO) were also used (Figure S2A). Western blot results showed that repeated sevoflurane treatment only increased AT8 (Treated vs. Control in WT mice; 1.41 ± 0.34 vs. 0.79 ± 0.24; *p* = 0.005) (Figure S2B,C), polyglutamylated Tubulin (Treated vs. Control in WT mice; 0.65 ± 0.12 vs. 0.43 ± 0.05; *p* = 0.03) (Figure S2D,E) and Spastin expression levels in WT (Treated vs. Control in WT mice; 1.14 ± 0.13 vs. 0.63 ± 0.15; *p* < 0.001) (Figure S2G) but not in Tau‐KO mice (Treated vs. Control in Tau‐KO mice; AT8: 0.10 ± 0.03 vs. 0.13 ± 0.16; *p* = 0.65; polyglutamylated Tubulin: 0.29 ± 0.09 vs. 0.28 ± 0.04; *p* = 0.80; Spastin: 0.46 ± 0.14 vs. 0.53 ± 0.11; *p* = 0.34) (Figure S2B–G). In the hippocampus of Tau‐KO mice, the absence of Tau did not lower the expression of TTLL6; no statistically significant difference in TTLL6 expression levels between control and sevoflurane group was found (Treated vs. Control in Tau‐KO mice; 0.97 ± 0.13 vs. 0.94 ± 0.18; *p* = 0.70) (Figure S2F). Furthermore, as demonstrated by immunostaining, repetitive sevoflurane administration did not promote TTLL6 fluorescence in the SR area of the CA3 region in Tau‐KO mice (Figure S2H,I).

The results indicate that TTLL6 plays a significant role in tubulin polyglutamylation and Spastin expression. However, the translocation of TTLL6 from axons to dendrites depends on the mislocalization of Tau.

### 3.5 TTLL6 Deficiency Mitigated the Overexpression of Spastin, Synaptic Plasticity, and Pre‐Adolescent Cognitive Impairments in Repetitive‐Anesthetized Neonatal Mice

**FIGURE 5:**
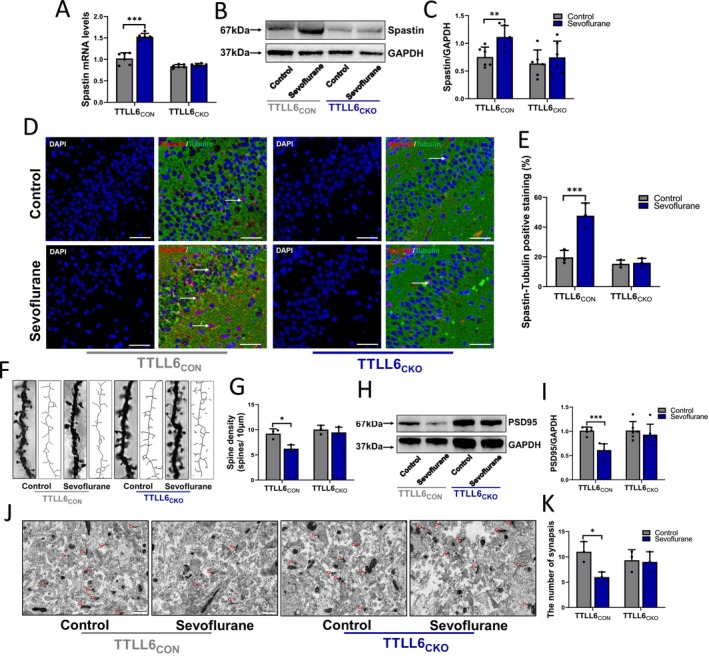
Effects of repeated sevoflurane exposure on Spastin expression and synaptic plasticity functions in TTLL6_CON_ or TTLL6_CKO_, neonatal mice. (A) Spastin mRNA and (B) Spastin protein expression and (C) qualification in the hippocampus after control or repetitive sevoflurane treatment; *n* = 6 mice/group. (D) Immunofluorescence of the protein labeled by Spastin (red) and α‐tubulin (green) in the CA3 area of hippocampus after control or repetitive sevoflurane treatment, Spastin‐α‐tubulin positive staining (pink) was marked with write arrows, scale bar: 50 μm, blue is DAPI. (E) Qualification of Spastin‐α‐tubulin positive staining (%) in different groups; *n* = 3 biological replicates. (F) Representative original photomicrographs and skeletonized dendrites images, and (G) qualification of Golgi‐stained dendrites in the CA3 region, *n* = 3 mice/group. (H) PSD95 protein expression and (I) qualification in the hippocampus after control or repetitive sevoflurane treatment; *n* = 6 mice/group. (J) Electron microscope showing the number of synapses (red triangles mark the postsynaptic density (PSD), serving as a marker for synapse counting), scale bar: 1 μm. (K) Qualification of synapsis numbers, *n* = 3 mice/group. *p < 0.05, **p < 0.01, ***p < 0.001.

RT‐PCR and western blot results showed that repetitive sevoflurane treatment enhanced both Spastin mRNA (Treated vs. Control in TTLL6_CON_ mice; 1.53 ± 0.07 vs. 1.02 ± 0.13; *p* < 0.001) (Figure 5A) and protein expression levels (Treated vs. Control in TTLL6_CON_ mice; 1.11 ± 0.21 vs. 0.75 ± 0.18; *p* = 0.009) (Figure 5B,C) in TTLL6_CON_ mice, and immunostaining results revealed that repetitive sevoflurane treated‐TTLL6_CON_ mice had more Spastin‐Tubulin positive staining in the CA3 area of the hippocampus than those in control mice (Treated vs. Control in TTLL6_CON_ mice; 47.98% ± 8.42% vs. 20.07% ± 4.53%, *p* = 0.007) (Figure 5D,E). However, no difference in Spastin mRNA and protein expression between sevoflurane and control groups in TTLL6_CKO_ mice was found (Figure 5A–E).

Research on dendritic spines and synapses of the cerebral hippocampus in the CA3 region showed that in TTLL6_CON_ but not in TTLL6_CKO_ mice, compared with control conditions, repeated sevoflurane treatment decreased dendritic spine density (Treated vs. Control in TTLL6_CON_ mice; 6.23 ± 0.76 vs. 9.22 ± 0.96; *p* = 0.01) (Figure 5F,G), lowered the expression of PSD95 (Treated vs. Control in TTLL6_CON_ mice; 0.61 ± 0.13 vs. 1.01 ± 0.07; *p* < 0.001) (Figure 5H), and reduced the number of synapses (Treated vs. Control in TTLL6_CON_ mice; 6.00 ± 2.00 vs. 11.00 ± 2.00; *p* = 0.02) (Figure 5J,K).

**FIGURE 6:**
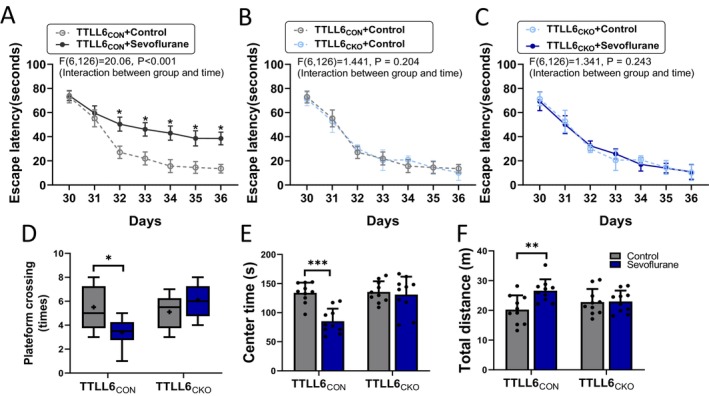
Effects of repeated sevoflurane exposure during the neonatal stage on cognitive functions in TTLL6_CON_ or TTLL6_CKO_ pre‐adolescent mice. Behavioral tests were conducted from P30 to P36 (MWM test: P36; MWM training: P30‐P36; open‐field test: P30). Qualification of escape latency (P30‐P36) (A) between Control and Sevoflurane groups in TTLL6_CON_ mice, (B) between Control in TTLL6_CON_ mice and Control in TTLL6_CKO_ mice, and (C) between Control and Sevoflurane groups in TTLL6_CKO_ mice. Qualification of (D) platform crossing number (P36) in MWM, (E) centre time, and (F) total distance in the open‐field test after control or repetitive sevoflurane treatment; *n* = 10 mice/group. *p < 0.05, **p < 0.01, ***p < 0.001.

Finally, conditional knockout of TTLL6 in the hippocampus and cortex attenuated the repetitive neonatal sevoflurane exposure‐induced spatial learning and memory abilities in pre‐adolescent mice (Treated vs. Control in TTLL6_CKO_ mice; Escape lantency: *F* = 1.34; *p* = 0.24; Plateform crossing times: 6.00 [2.50] vs. 5.50 [2.50]; *p* = 0.18) (Figure 6C) and anxiety‐like behavior in pre‐adolescent mice (Treated vs. Control in TTLL6_CKO_ mice; Center time: 131.20 ± 30.56 s vs. 135.60 ± 18.34 s; *p* = 0.70; Total distance: 22.97 ± 3.69 m vs. 22.79 ± 4.39 m; *p* = 0.92) (Figure 6E,F).

The findings in Figures 4, 5, 6 suggest that TTLL6 deficiency can reduce tubulin polyglutamylation, Spastin overexpression, synaptic plasticity, and cognitive impairment caused by excessive Tau phosphorylation and missorting induced by repetitive sevoflurane administration.

## 4 Discussion

Numerous studies have demonstrated that elevated levels of Tau in neonatal mice can lead to cognitive and synaptic plasticity impairments in the developing brain caused by repetitive sevoflurane administration [15, 31, 32, 33, 34]. However, the underlying mechanism remains unclear. Previous research has predominantly focused on various phosphorylation sites of the Tau protein, attempting to mitigate cognitive impairment caused by repeated sevoflurane exposure by modulating one of these phosphorylation sites [32, 33, 34]. Despite these efforts, the exact phosphorylation sites have yet to be definitively identified. In the current research, we found that repeated sevoflurane treatment increased microtubule‐unbound Tau (MUT) levels, and a large number of MUT may missort from the axon into the somatodendritic compartment, further inducing the mislocalization of TTLL6 into the dendrites. As a microtubule‐modifying ligase, enhanced dendritic TTLL6 may catalyze the addition of polyGlu residues to Glu residues at the C‐terminal tail of α‐tubulin, resulting in tubulin polyglutamylation, Spastin overexpression, and overactivation. Blocking TTLL6 expression reverses repeated neonatal anesthesia‐induced cognitive and synaptic plasticity impairments in the developing brain. However, TTLL6 deficiency has no effect on the increase in Tau phosphorylation generated by repeated sevoflurane exposure; nonetheless, the Tau gene knockout can reduce TTLL6 dendritic missorting. Thus, TTLL6 is transferred from somatic cells to dendrites via a Tau‐dependent mechanism. The conditional knockout of TTLL6 with a specific deletion in hippocampal and cortical cells can reduce synaptic plasticity and cognitive impairment caused by excessive Tau phosphorylation induced by repetitive sevoflurane administrations. MUT likely contributes to the mechanisms of repetitive sevoflurane‐induced early neurotoxicity in the developing brain through a Spastin‐related microtubule disruption caused by TTLL6 dendritic missorting.

Dendritic spines are spiky projections of the postsynaptic membrane on the surface of dendrites and serve as the structural foundation for neuronal connections and signal transduction in the nervous system. They receive most of the excitatory stimuli entering the brain [35]. Changes in synaptic plasticity usually occur along with morphological changes, such as dendritic spine formation, dissociation, expansion, and atrophy [10]. Moreover, microtubules continuously enter dendritic spines during neuronal activity, contributing to the composition of the cytoskeleton of dendritic spines, causing the heads of dendritic spines to expand (forming mushroom‐shaped and short, thick spines), promoting dendritic spine maturation, and increasing the content of PSD95 in dendritic spines, which plays an important role in memory formation [19, 20, 36]. Our previous study linked variations in dendritic spine density to cognitive performance [18, 30]. Here, repeated sevoflurane inhalation during the neonatal stage elicited anxiety‐like behavior and induced spatial learning and memory impairment in pre‐adolescent mice as shown by the results of the open‐field and MWM tests, respectively. Following repetitive sevoflurane anesthesia, the number of synapses and PSD95 levels in the CA3 area decreased significantly (Figure 1). Therefore, repeated sevoflurane treatment may cause cognitive impairment in pre‐adolescent mice through the regulation of dendritic spine density, maturity, and activity in the CA3 area of the hippocampus. This issue requires further investigation.

Subsequently, the potential reasons for repetitive sevoflurane‐induced microtubule shear and decreased dendritic spine density were examined. To date, three microtubule‐cleaving enzymes have been identified: Spastin, Katanin, and Fidgetin [37]. Among these, Spastin, which is encoded by SPG4 and discovered through mutations in patients with hereditary spastic paraplegia, is a microtubule‐cleaving protein that is currently the subject of extensive research. This protein contributes to the production of collaterals, the growth of neuronal processes, and changes in the architecture and function of dendritic spines. Additionally, Spastin plays a significant role in neural development and may be related to the regulation of microtubules [26, 38, 39]. For instance, the percentage of mature dendritic spines in neurones can be markedly decreased when the Spastin gene is overexpressed [40]. Compared to control conditions, repeated sevoflurane treatment enhanced the levels of Spastin in primary neurones and neonatal mice, while decreasing ATP levels. Additionally, in the brain tissues of neonatal mice, repeated sevoflurane inhalation decreased the expression levels of phosphorylated Spastin at threonine 598 and serine 595 but increased it at serine 99 and serine 101 (Figure 2; and Figure S1). When combined, repetitive sevoflurane exposure increased dendritic Spastin activation and levels, which may account for increased microtubule disruption in the developing brain. Spastin activation requires a significant quantity of ATP and may be induced by variations in Spastin phosphorylation at different sites.

Aberrant microtubule polyglutamylation can alter the microtubule shearing rate, affecting axonal transport and resulting in neurotoxicity [25, 38]. The TTLL6, a member of the TTLL family, is a kind of MAP that modulates MT transport and the binding of other MAPs to MT by adding glutamate residues to the C‐terminus of α‐ microtubule proteins, catalyzing microtubule polyglutamylation [41]. Furthermore, Tau is another type of MAP, and our previous studies demonstrated that increased expression of phosphorylated Tau and MUT is strongly associated with repetitive sevoflurane exposure‐induced neurotoxicity in the developing brain of neonates [15, 16, 17]. According to cell tests for AD, Tau and TTLL6 proteins interact in the structural domain, which might produce neurotoxicity in vitro [22, 42]. Our current study showed that repeated sevoflurane administration elevated MUT and pTau levels, promoted Tau‐TTLL6 binding, improved Tau and TTLL6 somatodendritic missorting, and ultimately boosted polyglutamylated Tubulin expression in the hippocampal tissue or neurons of neonatal mice (Figure 3). This finding suggests that Tau and TTLL6 may be key targets for understanding the mechanism of cognitive impairment and neurotoxicity in the developing brains of neonatal mice following repetitive sevoflurane inhalation.

To investigate the critical role of Tau and TTLL6 in repetitive sevoflurane anesthesia‐induced cognitive and synaptic plasticity impairments, WT, Tau‐KO, TTLL6_CKO_, and TTLL6_CON_ mice were used in subsequent experiments. The results indicated that knocking out Tau expression in neonatal mice could reverse the increase in Spastin and polyglutamylated Tubulin levels, the decrease in PSD95 production, and the enhancement of TTLL6 somatodendritic missorting caused by repetitive sevoflurane administration (Figure S2). Translocation of TTLL6 from axons to dendrites also depends on Tau sorting. Furthermore, repeated sevoflurane treatment did not increase polyglutamylated Tubulin or Spastin expression in TTLL6_CKO_ mice as it did in TTLL6_CON_ mice. Although repetitive sevoflurane treatment resulted in a high expression of phosphorylated Tau in TTLL6_CKO_ mice, TTLL6 deficiency might attenuate Tau‐dependent synaptic plasticity abnormalities and cognitive impairment induced by sevoflurane. Thus, TTLL6 plays an important role in neurocognitive impairment induced by Tau overexpression under repetitive sevoflurane conditions (Figures 4, 5, 6). These findings indicate that TTLL6 is a critical factor in repetitive sevoflurane exposure‐induced dendritic spine remodeling and cognitive impairment in the developing brain of neonatal mice, with tau being the upstream factor and TTLL6 being a crucial target for neurotoxicity produced by repeated sevoflurane inhalation.

Our study has several limitations. First, while the neonatal mouse model is a valuable preclinical tool for studying age‐dependent neurotoxicity induced by multiple sevoflurane exposures, there are notable physiological and developmental differences between mice and humans, particularly in terms of brain development. Therefore, further research is needed to determine its relevance to young children. Second, due to technical limitations, this study could not directly capture images of microtubule invasion into dendritic spines under an electron microscope, necessitating further verification. Third, although our study provides insights into the role of TTLL6, the precise mechanisms by which it functions remain to be fully elucidated. Future research should focus on tracking the translocation of TTLL6 from axons to dendrites to deepen our understanding of its role in repeated anesthesia‐induced neurotoxicity and potential therapeutic targets.

## 5 Conclusions

In conclusion, repeated sevoflurane treatment during the neonatal stage may lead to cognitive impairment in pre‐adolescent mice by altering dendritic spine density, maturity, and activity in the CA3 area of the hippocampus in the developing brain. TTLL6 is a critical factor in Tau missorting‐induced tubulin polyglutamylation and Spastin overexpression, with Tau being an upstream regulator. TTLL6 serves as a crucial target for neurotoxicity produced by repetitive sevoflurane treatment (Figure S3).

## Author Contributions

**Yang Yu:** conceptualization, investigation, writing – original draft, funding acquisition. **Yue Zhao:** methodology, formal analysis, resources, data curation. **Jingyu Feng:** methodology, data curation, writing – original draft. **Naqi Lian:** methodology. **Jiafeng Yu:** data curation. **Yongyan Yang:** formal analysis. **Junyan Yao:** writing – review and editing, supervision. **Yonghao Yu:** writing – review and editing, supervision, funding acquisition.

## Disclosure

Transparency statement: The design, data collection, analysis, and interpretation study adhere to rigorous scientific standards. All relevant data and methodologies have been clearly described in this manuscript, and any reproducible data are available upon request. There has been no selective reporting, and all hypotheses were based on a prespecified analysis plan.

## Conflicts of Interest

The authors declare no conflicts of interest.

## Supporting information


Data S1.

## Data Availability

All data relevant to the study are included in the article and its Supporting Information, which are available from the corresponding authors upon reasonable request.
