# Supplementary material for: Repeated Exposure to Sevoflurane in Neonatal Mice Induces Cognitive and Synaptic Impairments in a TTLL6‐Mediated Tubulin Polyglutamylation Manner
Source: CNS Neurosci Ther. 2025 Apr 9;31(4):e70376. doi: 10.1111/cns.70376 (PMC11979716; doi:10.1111/cns.70376)
Supplement: Supplementary file 1 — Data S1. [file CNS-31-e70376-s001.zip › cns70376-sup-0002-Supplemental File (unedited gels).pdf]

Full unedited gel/blot for Fig.1H

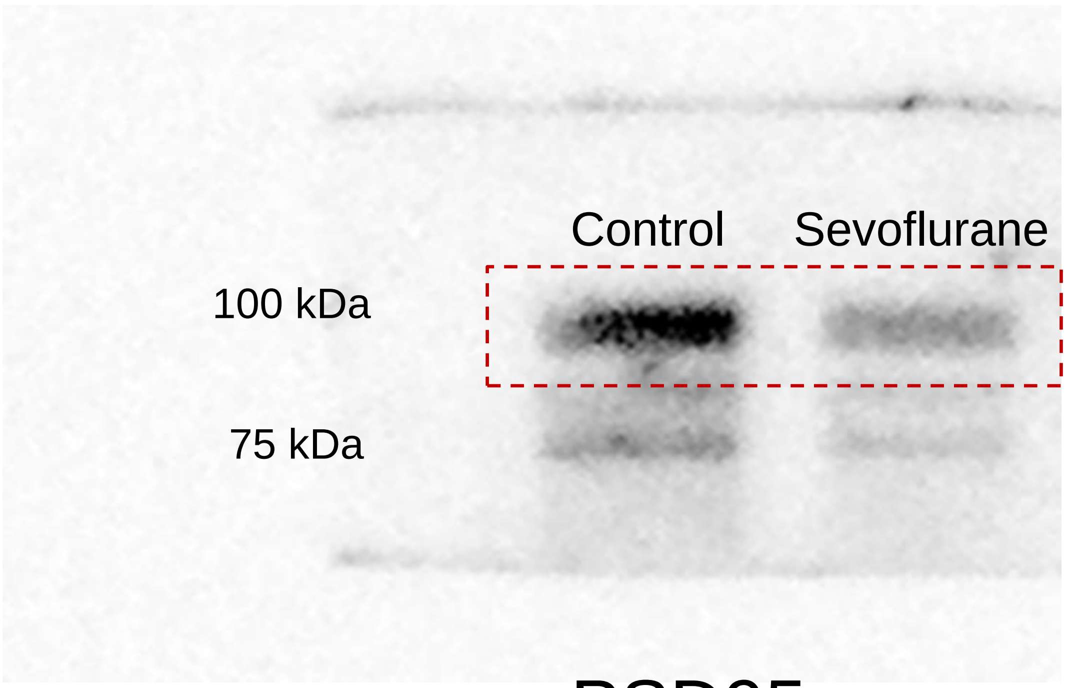

PSD95

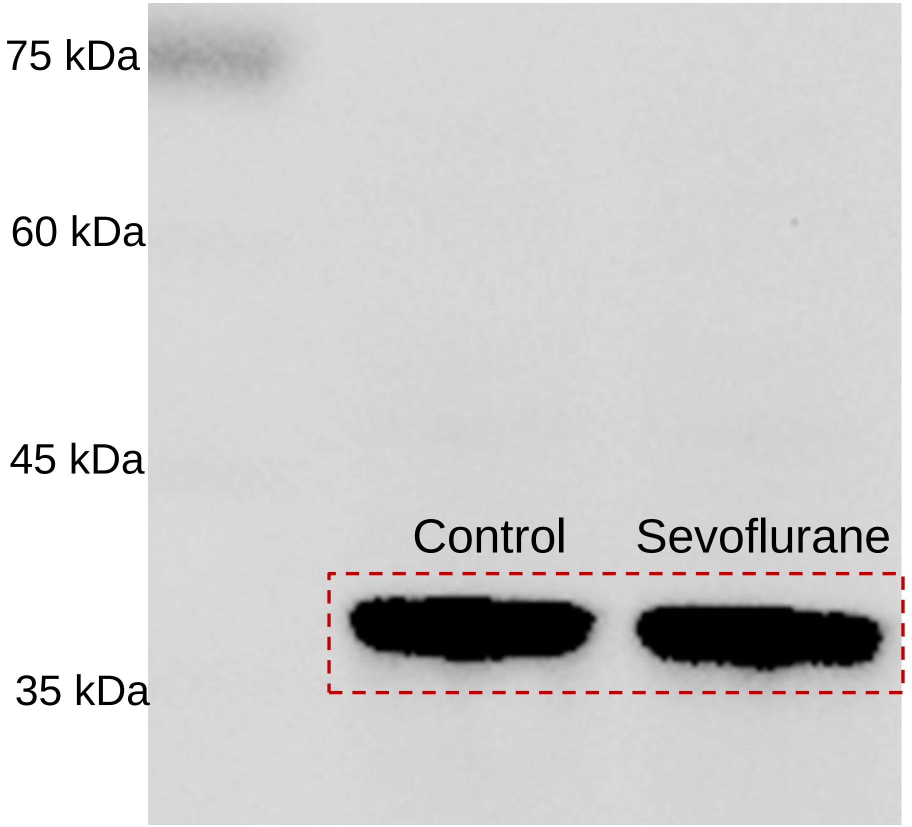

GAPDH

Full unedited gel/blot for Fig.2B

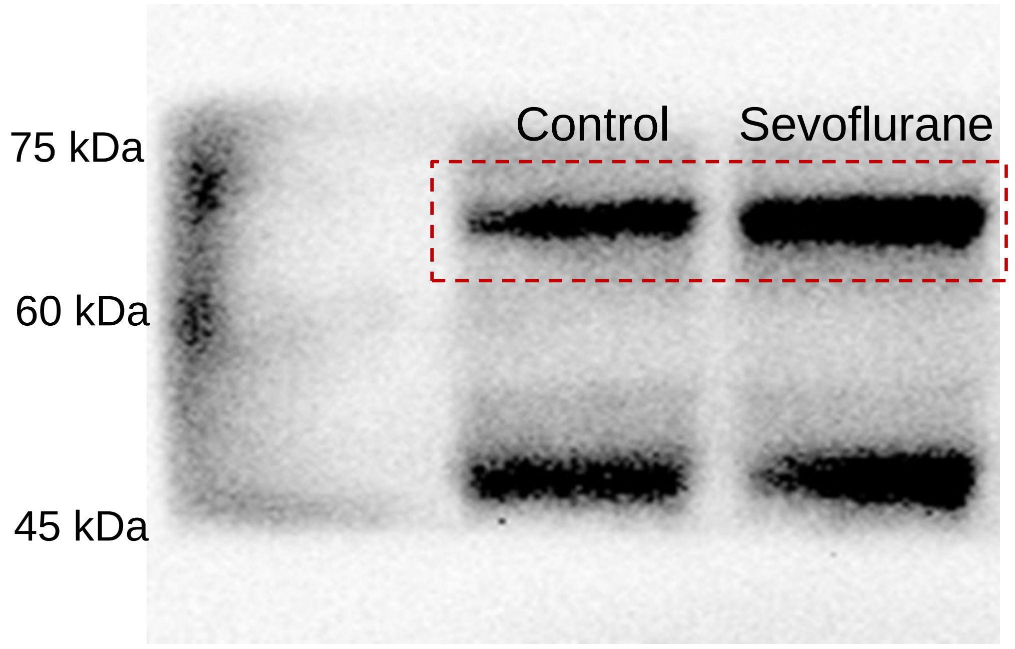

Spastin

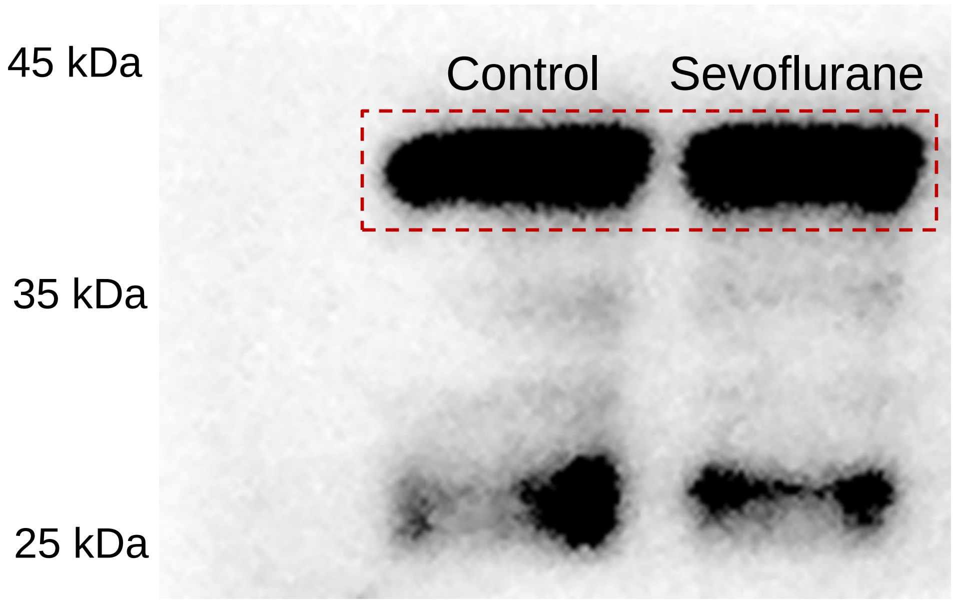

GAPDH

Full unedited gel/blot for Fig.3A

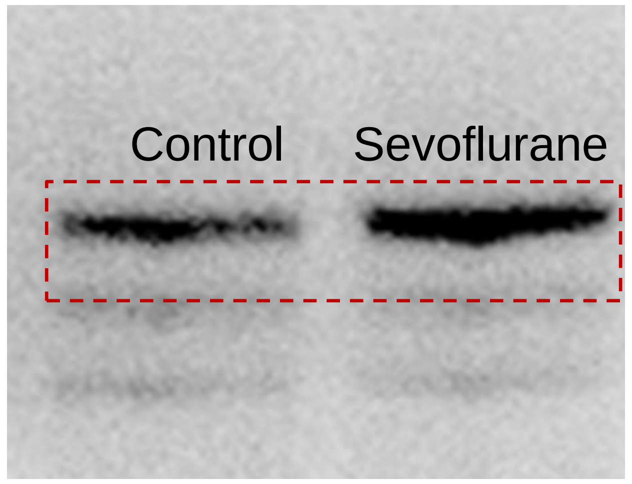

Polyglutamylated Tubulin

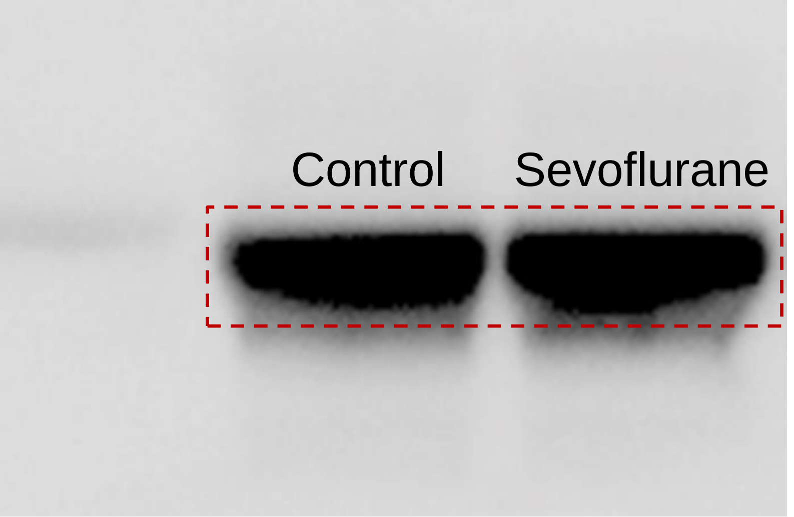

TTLL6

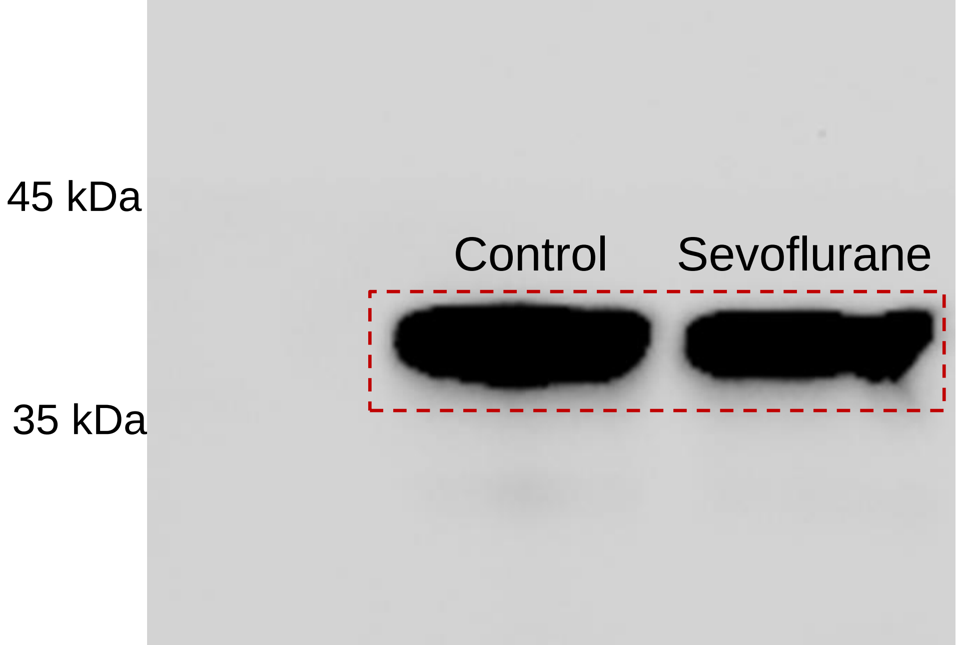

GAPDH

Full unedited gel/blot for Fig.3C

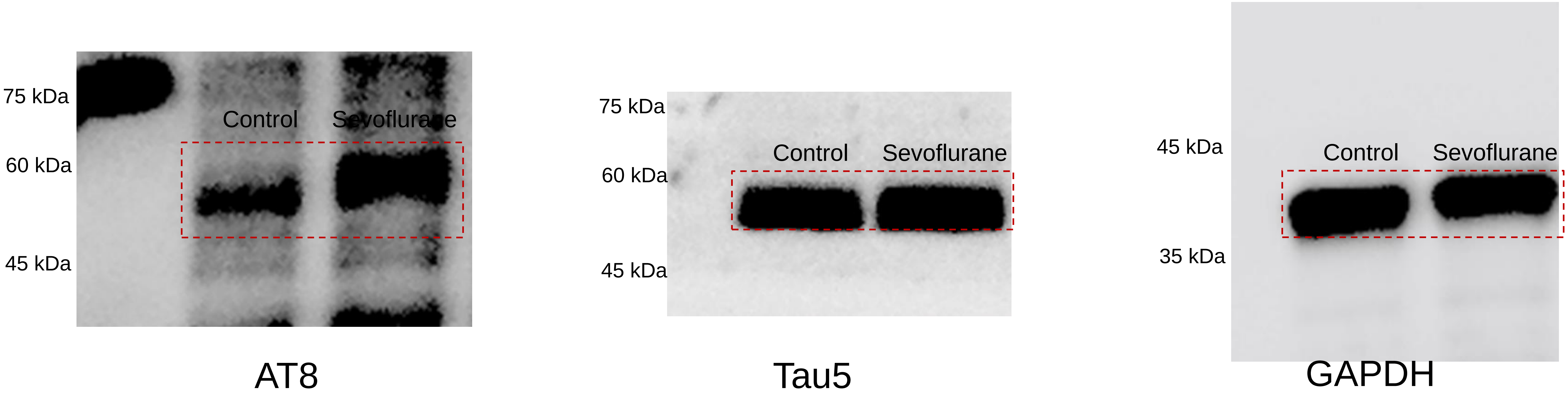

Full unedited gel/blot for Fig.3E

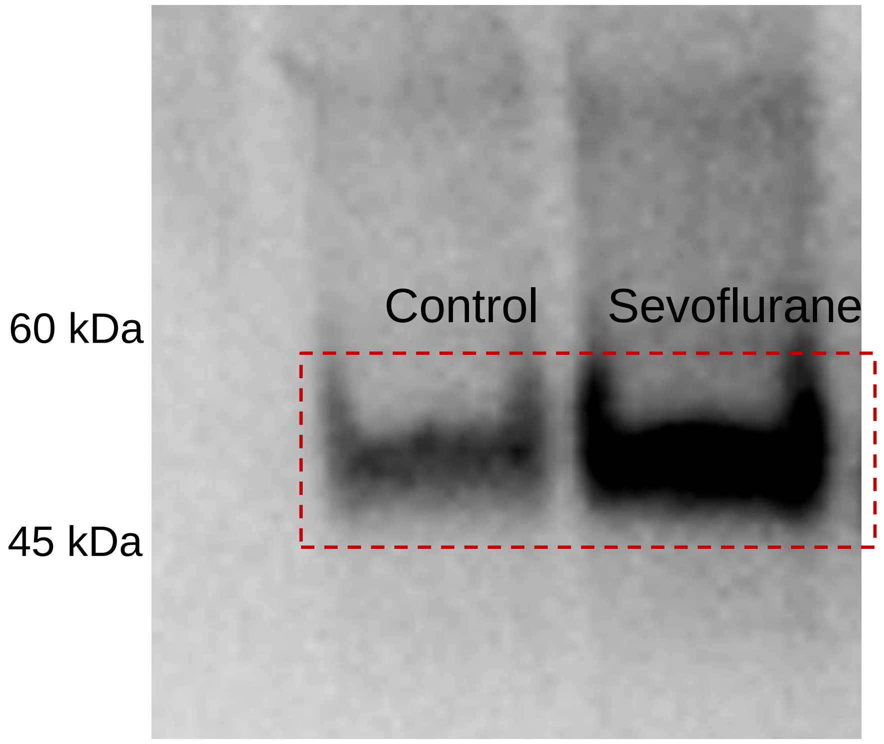

Tau (supernatant)

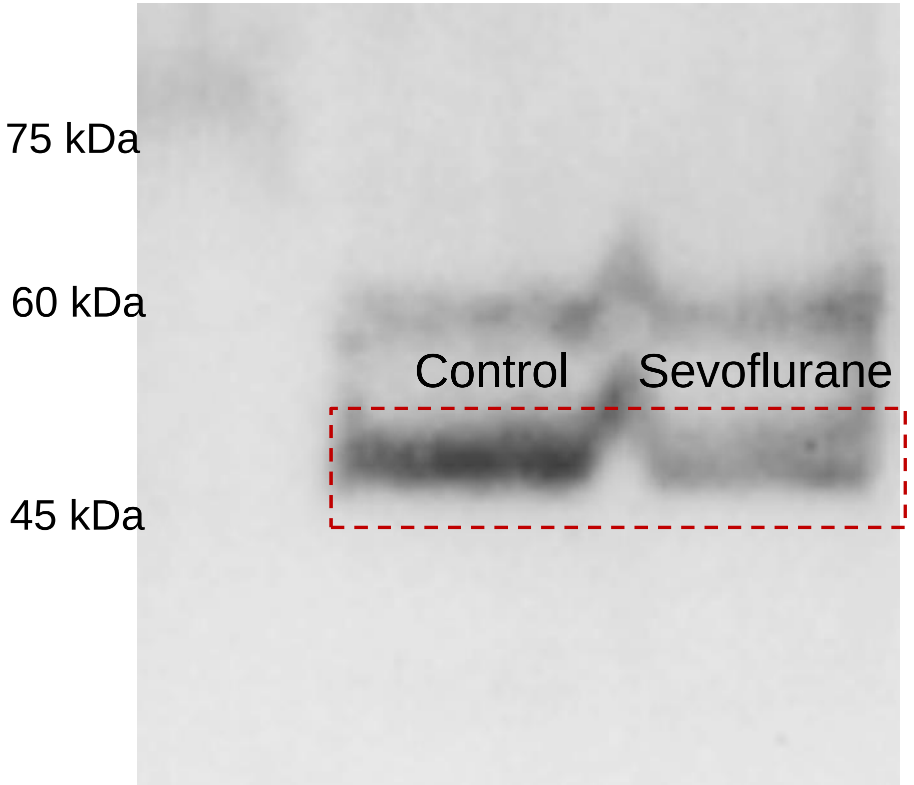

Tau (pellet)

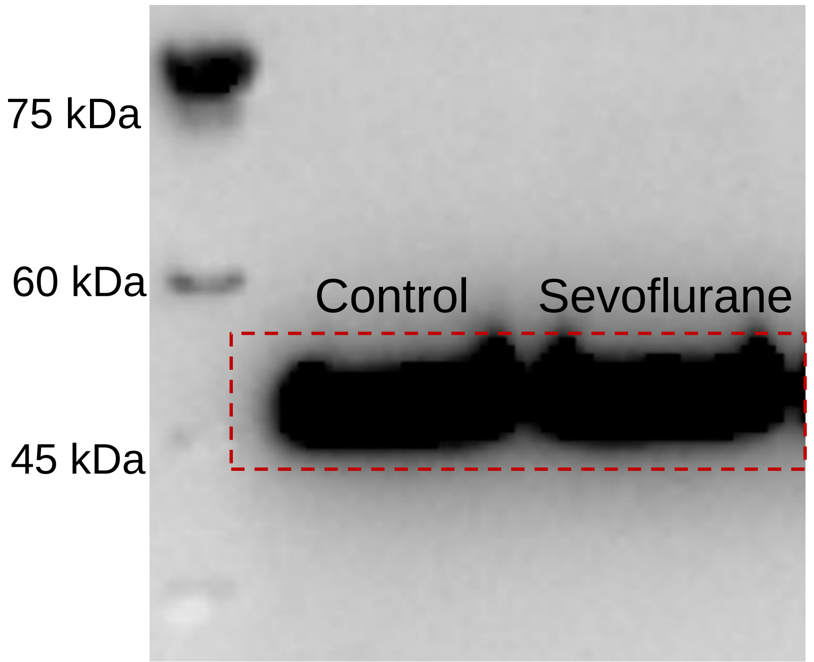

Tubulin (input)

Full unedited gel/blot for Fig.3H

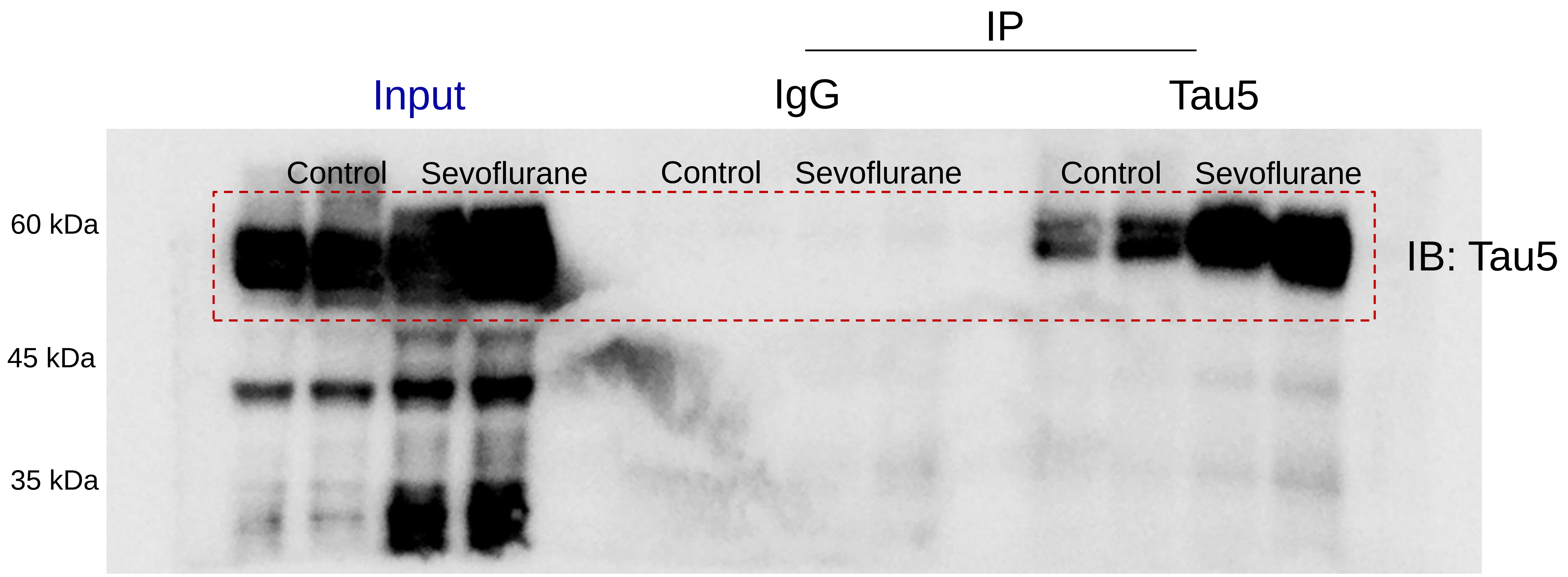

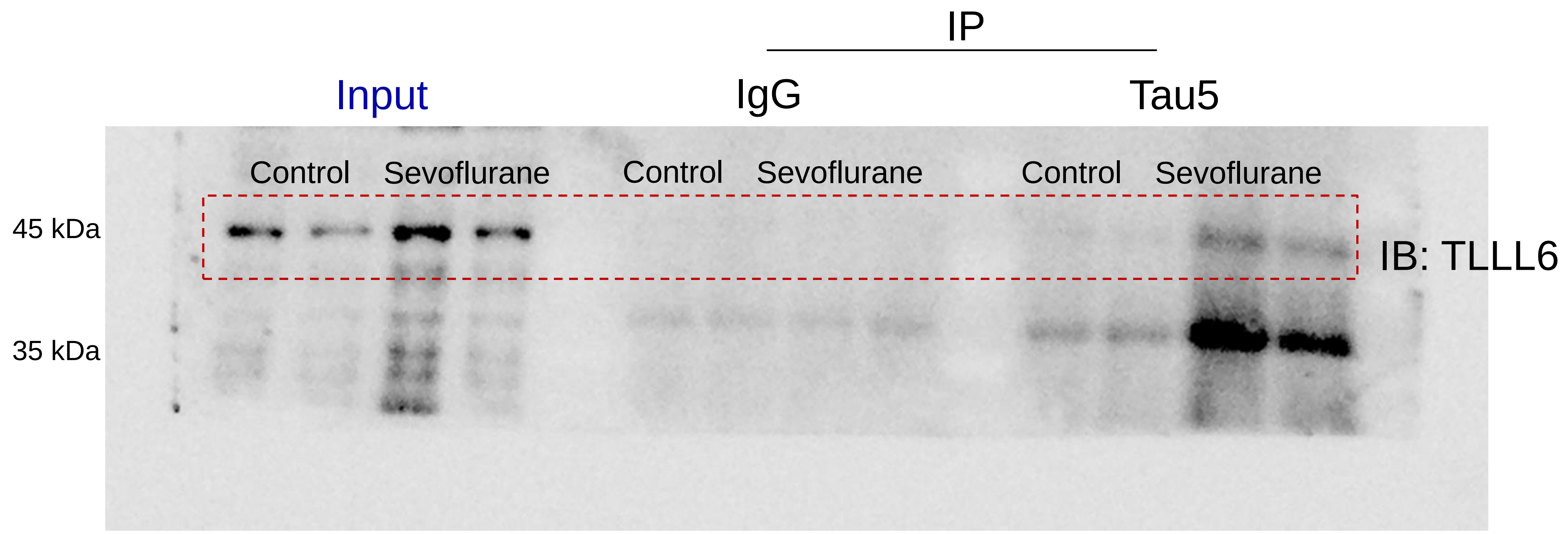

Full unedited gel/blot for Fig.4B

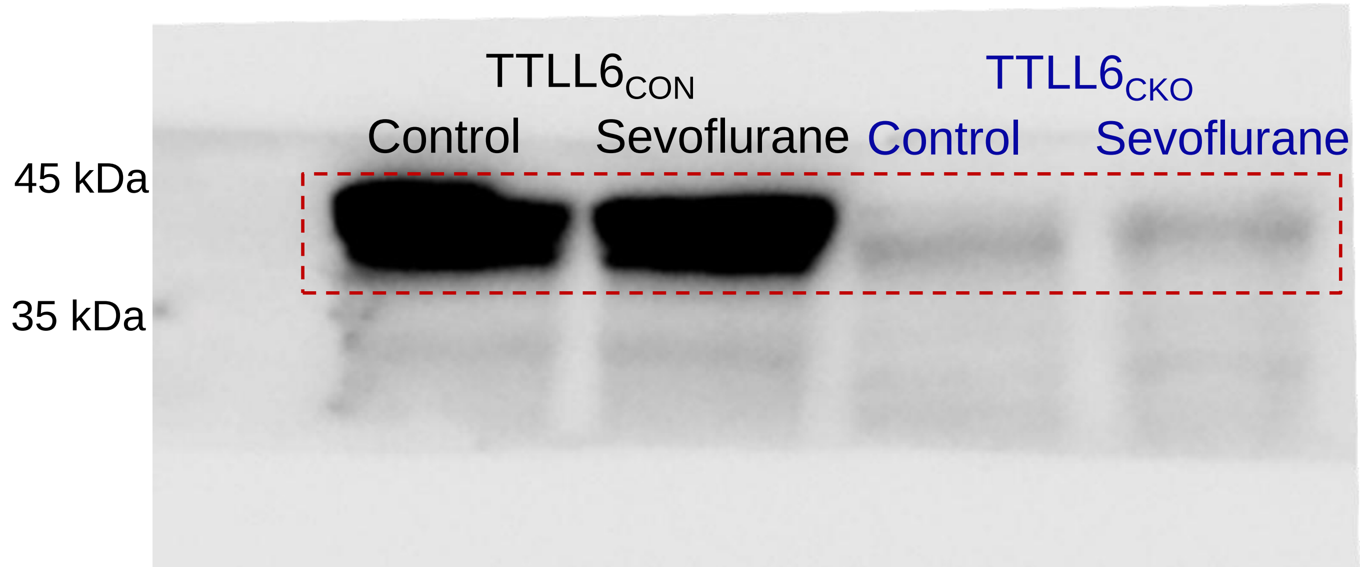

TTLL6

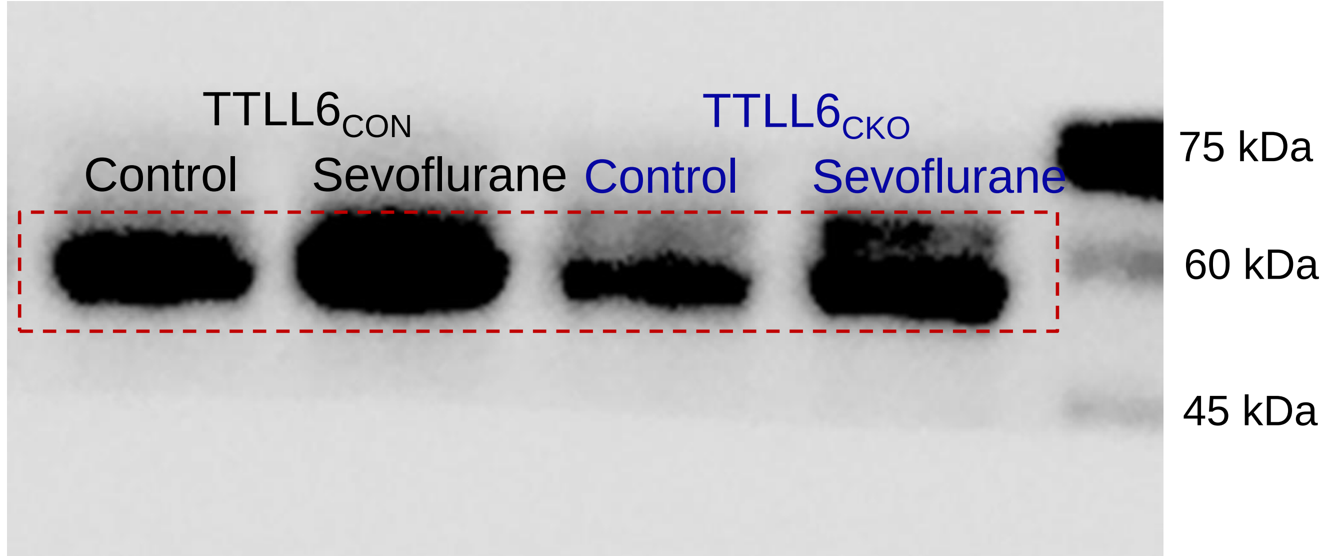

AT8

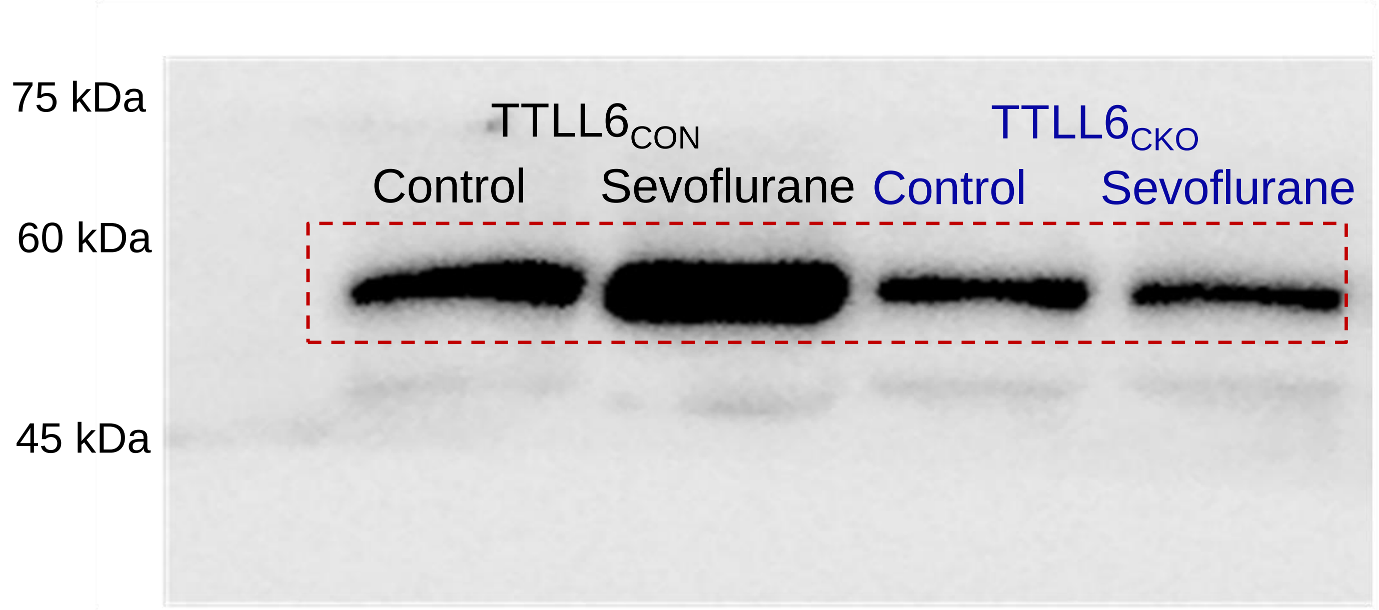

Polyglutamylated Tubulin

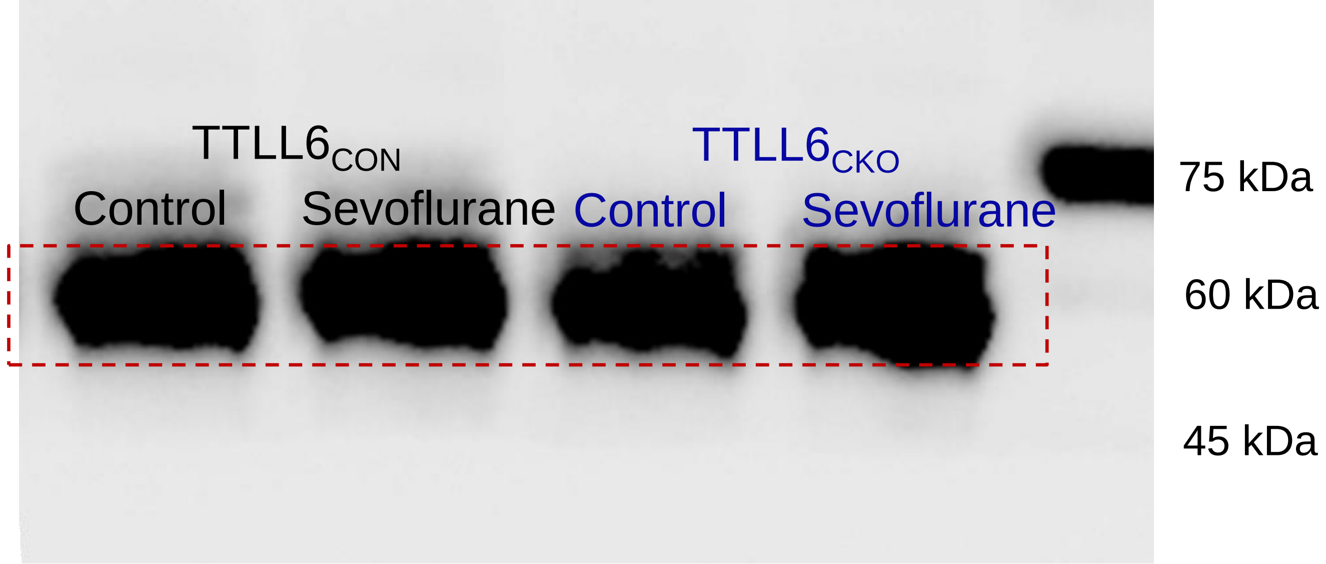

Tau5

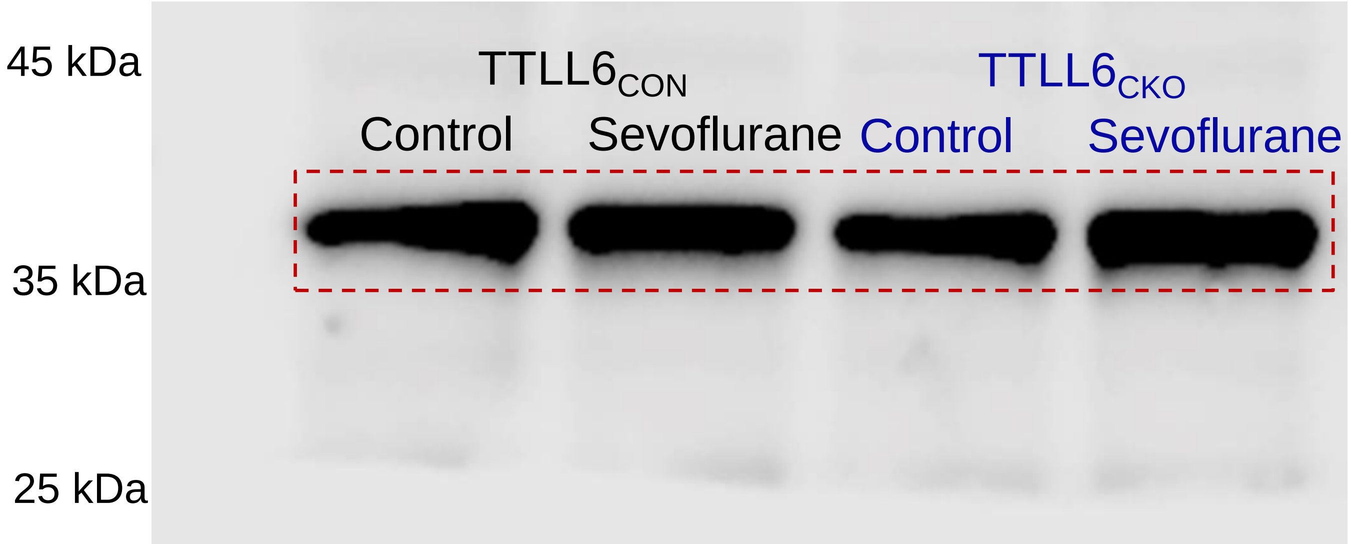

GAPDH

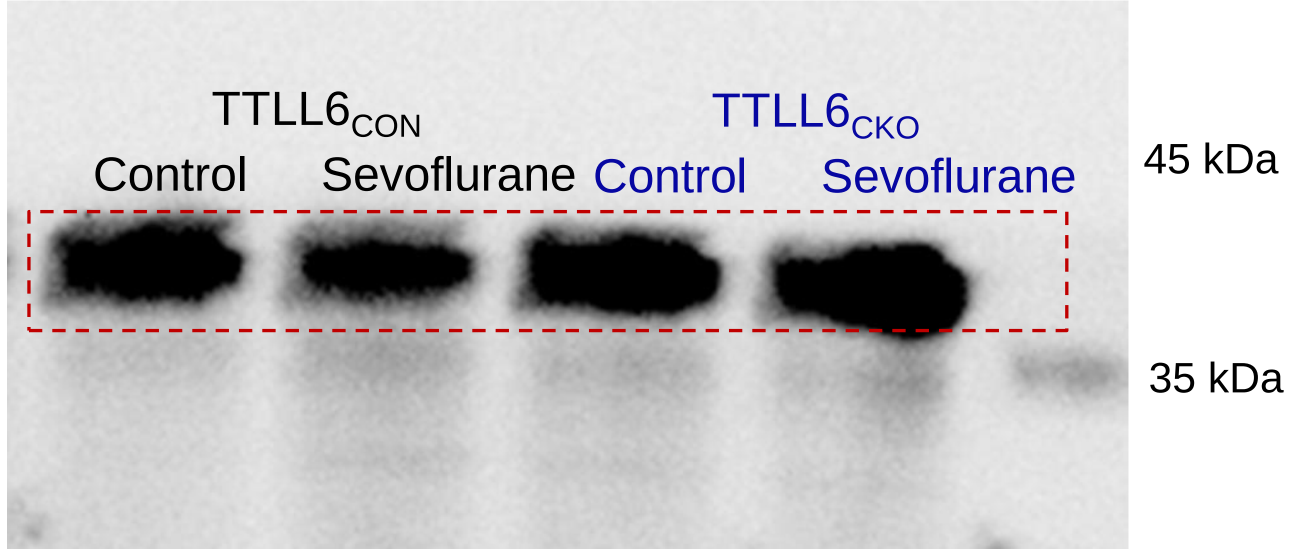

GAPDH

Full unedited gel/blot for Fig.5B

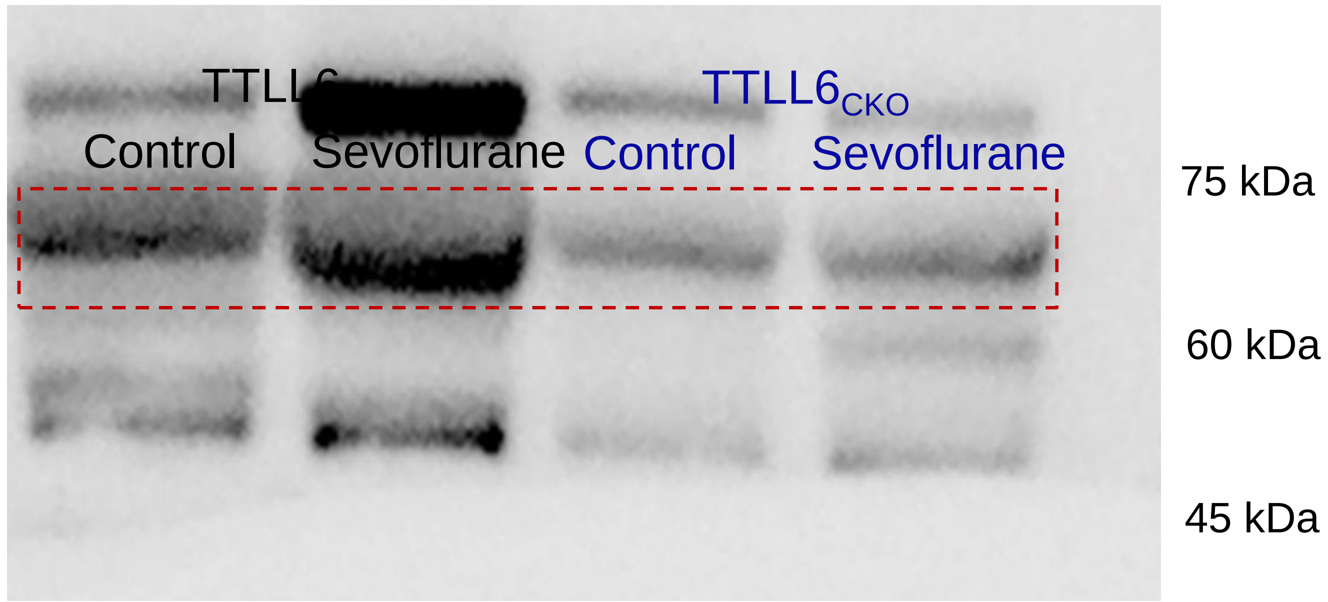

Spastin

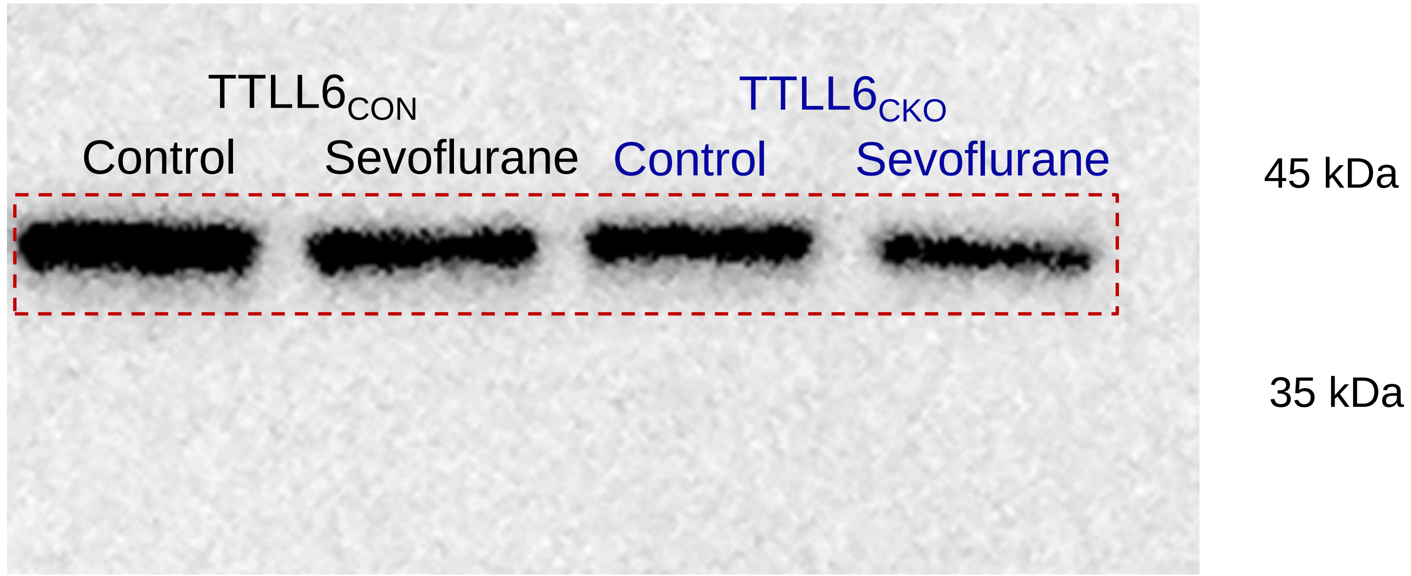

GAPDH

Full unedited gel/blot for Fig.5H

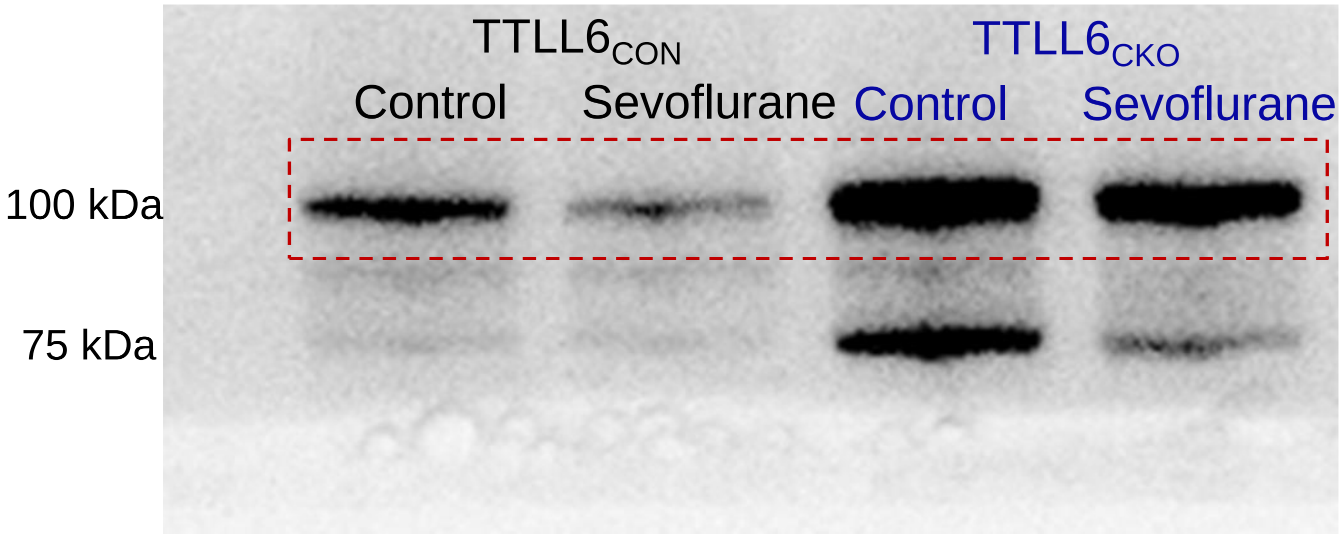

PSD95

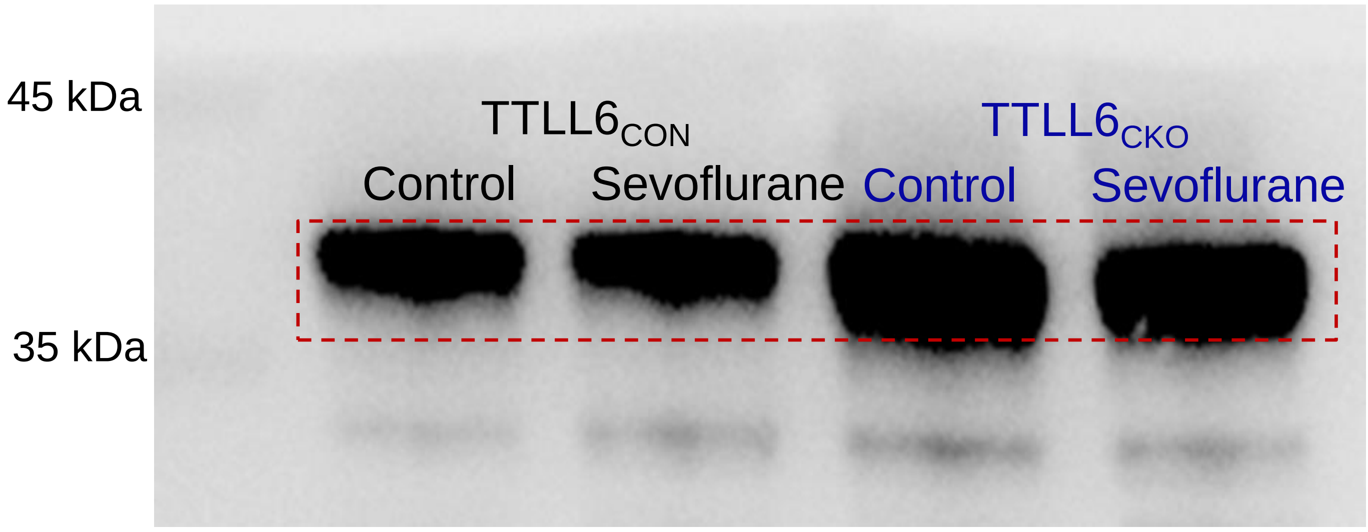

GAPDH
