# Supplementary material for: Repeated Exposure to Sevoflurane in Neonatal Mice Induces Cognitive and Synaptic Impairments in a TTLL6‐Mediated Tubulin Polyglutamylation Manner
Source: CNS Neurosci Ther. 2025 Apr 9;31(4):e70376. doi: 10.1111/cns.70376 (PMC11979716; doi:10.1111/cns.70376)
Supplement: Supplementary file 1 — Data S1. [file CNS-31-e70376-s001.zip › cns70376-sup-0003-Supplementary Material (revised).docx]

**Repeated exposure to sevoflurane in neonatal mice induces cognitive and synaptic impairments in a TTLL6-mediated tubulin polyglutamylation manner**

**Supplementary Materials and Methods**

**Materials and methods**

***Mice, anaesthesia and treatment***

The Animal Experimental Ethics Committee of Tianjin Medical University General Hospital in Tianjin, China approved the animal protocol (Approval No. IRB2020-DW-36). Minimising the use of animals in scientific research is imperative. Adult wild type C57BL/6J mice (WT) of 2-month-old were obtained from Sipeifu Bioscience (licence number, SCXK 2019-0010; Beijing, China). Adult TTLL6 floxed mice (TTLL6_CON_: TTLL6^f/f^), Camk2-Cre mice and Tau knockout mice (Tau-KO) were acquired from the Shanghai Model Organisms Centre, Inc. (licence number, SCXK 2019-0002; Shanghai, China). Male heterozygous Camk2-Cre mice were crossed with female TTLL6^f/f^ mice to obtain TTLL6 conditional knockout mice (TTLL6_CKO_: Camk2-Cre^+^; TTLL6^f/f^) with specific deletions in the hippocampal and cortical cells. Neonatal mice at postnatal day 6 were obtained from our own breeding and only female mice were used in the research. The genotype of the transgenic mice was determined by analysing genomic DNA obtained from their tails.

A total of 102 wild type, 102 TTLL6_CON_, 102 TTLL6_CKO_ and 18 Tau-KO, neonatal mice were utilized in the study. Neonatal mice were induced with a mixture of 60% O_2_ and 3% sevoflurane for 2 hours. The gas concentrations in the chambers were maintained at the set values, and mice rectal temperature was maintained at 37 ± 0.5 °C by controlling the temperature. The sevoflurane group received 3% sevoflurane and 60% O_2_ for 2 h/day on P6, P8, and P10, whereas the control group received only 60% O_2_ for 2 h/day on P6, P8, and P10 as described by Lu et al[1]. The experiments were conducted simultaneously on each day. After the modelling was completed, the mice were returned to their mother mouse cage once they exhibited stable breathing and were fully awake. Notably, blood gas values are not significantly affected by the use of 3% sevoflurane and 60% O_2_ during anaesthesia[2].

***Primary neurons culture and treatment***

Mouse embryos at 15 days of gestation were decapitated and euthanised in various experimental groups. Embryonic brains were dissected and immersed in 75% alcohol for a minute. Hippocampal tissue was removed and washed in precooled DMEM. The hippocampus was then dissected into small fragments using eye scissors and placed in a culture dish containing 2 ml of DMEM. Then, 2 ml of 0.25% Trypsin, an enzyme that aids protein breakdown, was added to each Petri dish. This mixture was incubated at 37 ℃ for 30 minutes to allow for digestion. Tissue debris and digestive fluid were transferred to a centrifuge tube, and the same volume of digestive terminating fluid (DMEM + 10% FBS, Gibco, USA) was added to gently blow tissue debris 15 times: each time, the tissue completely settled to the bottom of the tube, and it was blown the next time. After blowing, it was filtered through a sieve of 60 μm. After 1 min, the supernatant containing the single-cell suspension was collected and mixed with the appropriate digestive termination solution. The cells were counted under a microscope at a density of 70 ×10^5^/ml; 1.5 ml of this mixture was inoculated into each well of a 6-well Petri dish. After 4 h of culture, neuronal growth was observed by replacing the neuronal culture medium with Neurobasal-A + 2% B27 (Engreen, Beijing, China). The neuron culture medium was replaced every 3 days, and half of the solution was replaced each time.

On the 5th day, the neurones in sevoflurane group were placed in a sealed resin box (20 cm × 15 cm ×7 cm) and treated with 21% O_2_, 5% CO_2_ and 4.1% sevoflurane [2 minimum alveolar concentration (MAC)] for 4 hours in a 37 ℃ incubator as described by Lu et al.[1]. The control group was incubated with 21% O_2_ and 5% CO_2_ for 4 h under the same conditions. A variable anaesthetic gas monitor (Vamos; Drager Medical AG & Co. KgaA, Germany) was used to continuously monitor the concentrations of O_2_, CO_2_, and sevoflurane during anaesthesia or control conditions.

***Open-field test***

The open-field test was performed as described previously[3]. Briefly, each mouse was placed near the wall of a 38 × 42 cm open field, and its movements were observed for 10 min. The total distance travelled and the time spent in the centre (15 × 15 cm imaginary square) were recorded using an Any-maze (Stoelting, Wood

Dale, IL, USA). Between trials, the open-field arena was cleaned with 70% ethanol and wiped using paper towels.

***Morris water maze (MWM)***

The Morris water maze test was conducted at P30 for 7 days (P30-36) with four trials each day. The escape latency of the mice during reference training was measured. Details of the Morris water maze test have been described previously[2]. On the last day, the platform was removed from the pool, and each mouse was placed in the opposite quadrant for a 90-second swim trial. The number of platform crossings was recorded as an indicator of the spatial memory.

***Brain tissue harvest, lysis, and protein quantification***

The mice were decapitated after P10 sevoflurane anaesthesia, and their hippocampi were harvested. The collected brain tissues were used for western blotting, co-immunoprecipitation, and microtubule-binding assays. The harvested brain tissues were homogenised on ice by using immunoprecipitation buffer (M-PER® Mammalian Protein Extraction Reagent, Cat# 78501, Thermo Fisher Scientific, Waltham, MA) plus protease inhibitor cocktail (Cat# 11836170001, Sigma-Aldrich). The lysates were collected and centrifuged for 15 min at 13,000 rpm. A bicinchoninic acid protein assay kit (Pierce, Iselin, NJ, USA) was used to measure the total protein concentration at 1000 rpm.

***Reverse transcriptase polymerase chain reaction (RT-PCR)***

Spastin mRNA levels were measured and normalised to glyceraldehyde 3-phosphate dehydrogenase (GAPDH). Mouse Spastin primers (ID No. QT01040893), and mouse GAPDH primers (ID No. QT01658692). PCR involved incubation at 50 °C for 30 minutes and 95 °C for 15 minutes, succeeded by 50 cycles of 94 °C for 15 seconds, 55 °C for 30 seconds, 72 °C for 30 seconds, 95 °C for 15 seconds, and 55 °C for 15 seconds.

***Microtubule binding assay***

A microtubule-binding protein spin-down assay kit (Cat# BK029; Cytoskeleton, Denver, CO, USA) was used. Briefly, 10 μg protein with 20 μL microtubules were added into a clean centrifuge tube in each treatment condition, general Tubulin buffer plus taxol was used to adjust the final volume to 100 μL. The reaction mixture was then incubated at room temperature for 30 min. Then, 10 μL reactions in each treatment condition were used for western blotting as the “input fraction”. The rest 90 μL reactions in each treatment condition were layered onto a 200 μL of the taxol supplemented cushion buffer and centrifuged at 20,000× *g* for 60 minutes. The supernatant containing soluble Tubulin was collected from the top of the tube as the cytosol fraction, and the pellet containing microtubule polymers and proteins bound to microtubules was resuspended in 50 μL of 1× Laemmli sample buffer. Proteins in supernatant, pellet, and “input fraction” were loaded to Tris-Glycine gels and analysed by western blotting using an anti-Tau46 antibody (Cat# T9450, 55 kDa, 1:2000, Sigma-Aldrich, USA) or anti-Tubulin antibody (Cat# ab6161, 55 kDa, 1:1000, Abcam, Cambridge, MA).

***Western blot***

Total Tau and TTLL6 expression levels were detected using anti-Tau5 antibody (Cat# ab80579, 55 kDa, 1:1000, Abcam) and anti-TTLL6 antibody (Cat# DF12178, 55 kDa, 1:1000, Affinity Biosciences), respectively. Spastin antibody (Cat# ab244354, 67 kDa, 1:1000; Abcam) was used to measure Spastin protein expression levels. The AT8 antibody (Tau-PS202/PT205, Cat# MN1020, 55 kDa, 1:2000, Thermo Fisher Scientific) was used to detect Tau phosphorylated at its serine 202 and threonine 205 residues. The PSD95 antibody (Cat# ab13552, 100 kDa, 1:1000, Abcam) was used to measure PSD95 protein expression levels. α-Tubulin (polyglutamylated) antibody (Cat# T9822, 50 kDa, 1:1000, Sigma-Aldrich) was used to measure polyglutamylated Tubulin expression levels. Finally, an antibody specific for the protein GAPDH (Cat# AF7021, 37 kDa, 1:5000, Sigma, Affinity Biosciences) was utilized as a reference to determine variations in the total protein quantity during loading. The quantification of Western blot was performed using the method described previously[4].

***Co-immunoprecipitation***

Co-immunoprecipitation (CO-IP) experiments were performed using mouse hippocampal tissue proteins to investigate the interaction between Tau and TTLL6. Following protein extraction, total protein was incubated with Tau5 (Cat# ab80579, 4 μg, Abcam) and negative control mouse normal IgG1 (Cat# 5415S, 4 μg, Cell Signalling) according to the manufacturer's instructions (Cat# 22202-100, beaver) to form antigen-antibody complexes. The complexes were then immobilised on magnetic beads for separation and purification, and the coprecipitated proteins were identified by immunoblotting. Although the Co-IP data was not measured quantitatively, three independent experiments to gather the necessary information were performed.

***Golgi staining and dendritic spine density analysis***

Golgi staining was performed on P10 mice. Staining and analysis were performed using a Golgi staining reagent kit (Cat#: PK401, FD Neuro Technologies, USA), as described in our earlier study[4].

***Electron microscope***

The brain tissues from 10-day-old mice that were anaesthetised with sevoflurane were collected and preserved in 2.5% glutaraldehyde at 4 °C for 24 hours. The samples were then treated with 1% osmium tetroxide and washed with 0.1 M phosphate buffer. Following dehydration, infiltration, and embedding, 50 nm sections were prepared using a microtome. The sections were then stained with uranyl acetate and lead citrate, air-dried overnight, and examined under an electron microscope.

***Immunohistochemistry***

Phosphorylated Tau antibody (AT8, Cat# MN1020, 55 kDa, 1:2000, Thermo Fisher Scientific), TTLL6 antibody (Cat# DF12178, 1:100, Affinity Biosciences), Spastin antibody (Cat# ab244354, 1:100, Abcam), and α-Tubulin antibody (Cat# ab6160, 1:50, Abcam) were used to measure expression levels and distribution of these four proteins in the CA3 region of the hippocampal tissue and primary neurones. Immunofluorescence experiments were performed as described previously[5,6].

***Multiplexed quantitative mass spectrometry-based phosphoproteomics***

Whole cerebral tissues were collected after the termination of sevoflurane-induced anaesthesia. Multiplexed phosphoproteomics using mass spectrometry was conducted according to established protocols[2]. Peptides were identified by searching against a comprehensive protein sequence database, including all mouse ORF database protein sequences (downloaded on 01/14/2014), as well as those of recognised impurities. High- and low-resolution spectra of the phosphopeptide data were annotated and merged in two separate searches.

***ATP measurement***

The levels of ATP in the hippocampal and cortical tissues of mice were measured using the ATP Colorimetric/Fluorometric Assay Kit, in accordance with the protocol provided by the manufacturer (Cat# ab83355, Abcam) and the methods described in our previous studies[2].

***Statistics***

Data analyses were performed using GraphPad Prism (version 9.0) and SPSS statistical software (version 21.0). Data obtained from biochemistry studies, centre time of open field test, total distance of open-field test and escape latency of MWM test is presented as mean ± standard deviation (SD). The numbers of platform crossings in the MWM are presented as medians with interquartile ranges. The number of mice was 10 in each group for behavioural studies, 6 in each group for western blotting, PCR, and ATP measurements, 4 in each group for mass spectrometry studies, and 3 in each group for the microtubule-binding assay, co-immunoprecipitation, Golgi staining, electron microscopy, and immunohistochemistry. These numbers were selected based on the results of our previous studies[2,7]. The interaction between time and group factors was determined using a two-way ANOVA with repeated measurements to analyse the difference in learning curves (based on escape latency) between mice in the control group and those treated with anaesthesia in the MWM. A post-hoc Bonferroni test was used to compare the difference in escape latency between the control and anaesthesia groups on each day of the MWM. The Mann-Whitney U test was used to determine the difference in platform crossing times between sevoflurane anaesthesia and control conditions. There were no missing data for the variables of the open-field test (centre time and total distance) and MWM (escape latency and platform crossing times) during the data analysis. Finally, to compare the two groups for other biochemical data, the unpaired t-test (if the values were in a Gaussian distribution) or the Mann-Whitney test (non-Gaussian distribution) was applied. The normality of each group of data was assessed using the Shapiro-Wilk test. Statistical significance was expressed as P < 0.05.

**References**

1. H. Lu, N. Liufu, Y. Dong, et al., "Sevoflurane Acts on Ubiquitination-Proteasome Pathway to Reduce Postsynaptic Density 95 Protein Levels in Young Mice," *Anesthesiology* 127,no.6 (2017):961-975.

2. Y. Yu, Y. Yang, H. Tan, et al., "Tau Contributes to Sevoflurane-induced Neurocognitive Impairment in Neonatal Mice," *Anesthesiology* 133,no.3 (2020):595-610.

3. S. Han, C. Tai, R. E. Westenbroek, et al., "Autistic-like behaviour in Scn1a+/- mice and rescue by enhanced GABA-mediated neurotransmission," *Nature* 489,no.7416 (2012):385-390.

4. J. Feng, H. Lin, Y. Zhao, Y. Yang, X. Zhuang, Y. Yu, Y. Yu, "Tandem mass tag-based quantitative proteomic analysis of effects of multiple sevoflurane exposures on the cerebral cortex of neonatal and adult mice," *Front Neurol* 13,(2022):1056947.

5. Y. Yu, M. Yang, X. Zhuang, J. Pan, Y. Zhao, Y. Yu, "Effects of toxic apolipoprotein E fragments on Tau phosphorylation and cognitive impairment in neonatal mice under sevoflurane anesthesia," *Brain Behav* 12,no.8 (2022):e2702.

6. Y. Yu, M. Yang, X. Zhuang, J. Pan, J. Feng, J. Yu, Y. Yu, "Neurotoxic 18-kDa apolipoprotein E fragment production contributes to anesthetic sevoflurane-induced tau phosphorylation and neuroinflammation in vitro," *Hum Exp Toxicol* 41,(2022):9603271221102519.

7. Y. Yang, F. Liang, J. Gao, et al., "Testosterone attenuates sevoflurane-induced tau phosphorylation and cognitive impairment in neonatal male mice," *Br J Anaesth* 127,no.6 (2021):929-941.

**Supplementary figures**


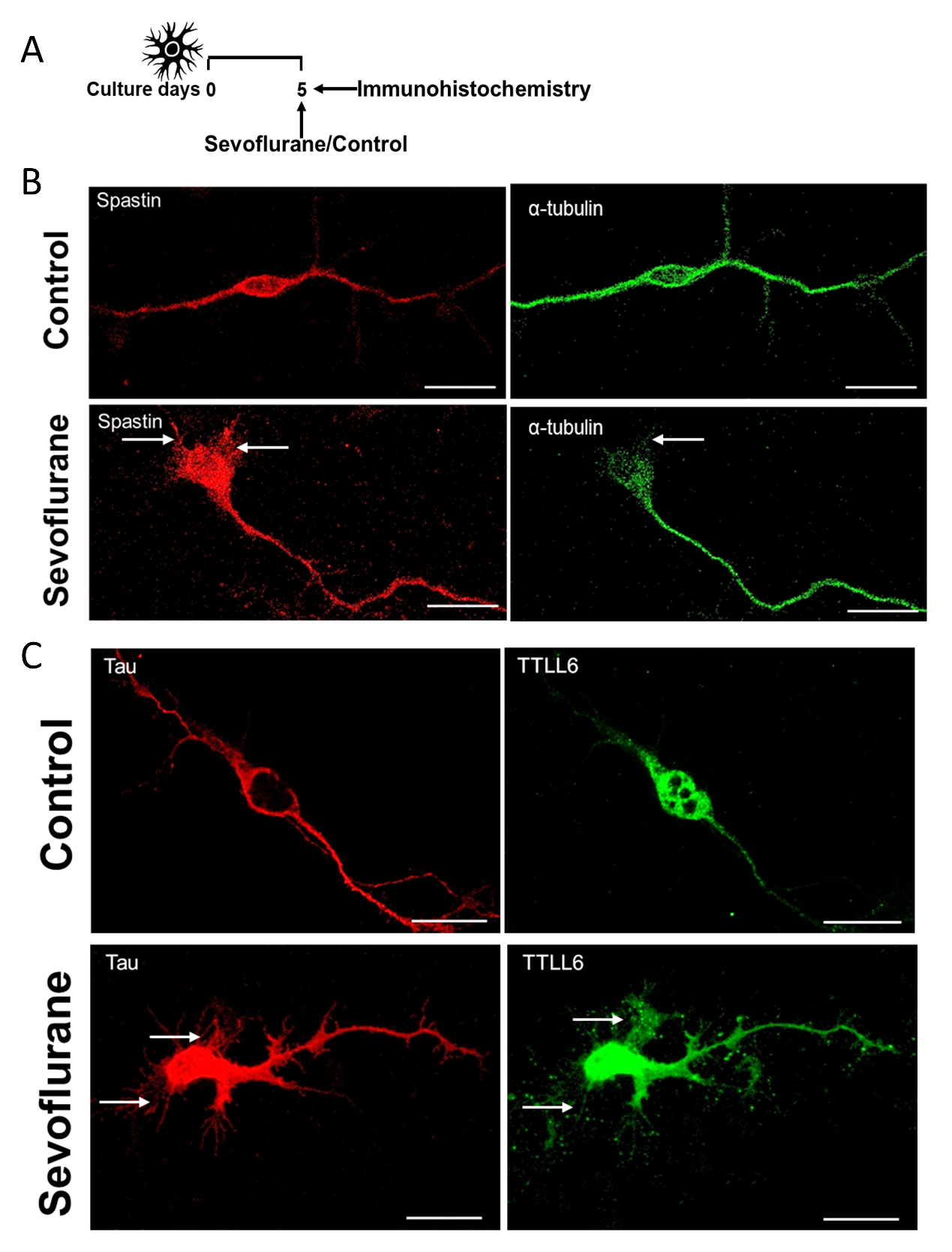


**Fig. S1. Effects of sevoflurane on Spastin, α-tubulin, Tau and TTLL6 expression in primary hippocampal neurons.** (A) Experimental design: After cultured 5 days, the mice hippocampus neuron cells were treated with treated with 21% O_2_, 5% CO_2_ and 4.1% sevoflurane for 4 hours or with 21% O_2_ and 5% CO_2_ for 4 h under the same conditions. (B) Immunofluorescence of the protein labelled by Spastin (red) and Tubulin (green) in the hippocampus neuron cells after control or sevoflurane treatment. Spastin expression levels increase in the dendrites (write arrows) when treated with sevoflurane, scale bar: 20 µm. (C) Immunofluorescence imaging showing the difference in Tau (red) and TTLL6 (green) expression in hippocampus neuron cells after treatment with control or sevoflurane; after sevoflurane treatment, Tau and TTLL6 protein expression levels increased and they to migrated to dendrites (write arrows), scale bar: 20 µm.


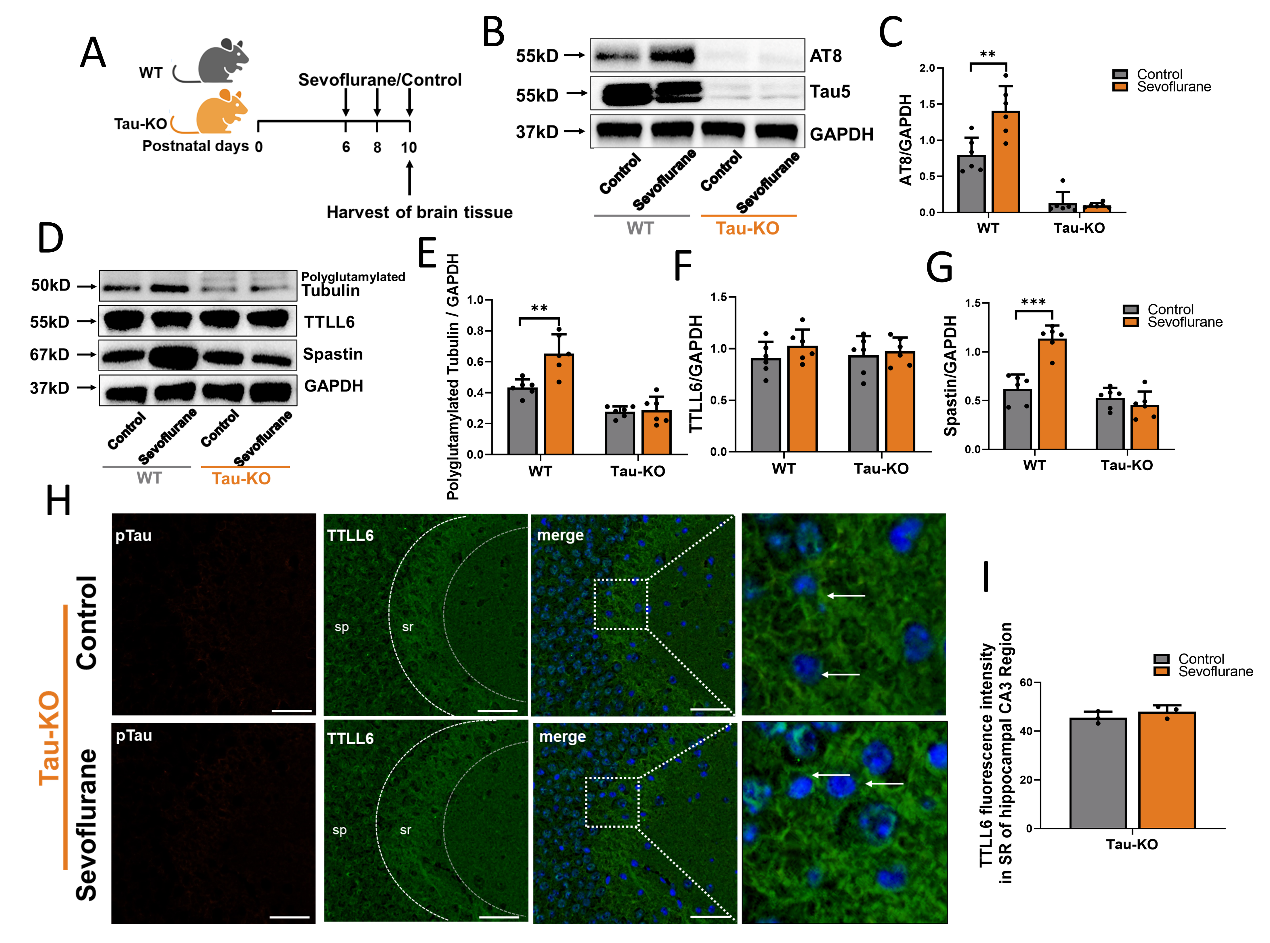


**Fig. S2. Effects of sevoflurane on related protein expression of Tau-TTLL6 missorting in WT and Tau-KO neonatal mice.** (A) Experimental design: WT and Tau-KO neonatal mice were administered 3% sevoflurane + 60% O_2_ or 60% O_2_ alone for 2 h every day for 3 days (P6, P8, P10). Hippocampus tissues were collected at the conclusion of the sevoflurane or control condition. (B) Expression of AT8 and Tau5 in the hippocampus after control or sevoflurane treatment. (C) Qualification of AT8 in different groups; n = 6 mice/group. (D) Expression of polyglutamylated Tubulin, TTLL6, Spastin in the hippocampus after control or sevoflurane treatment. Qualification of (E) polyglutamylated Tubulin, (F) TTLL6 and (G) Spastin in different groups; n = 6 mice/group. (H) Immunofluorescence of the protein labelled by pTau (red) and TTLL6 (green) in the CA3 area of hippocampus after control or sevoflurane treatment in Tau-KO mice. pTau-TTLL6 co-expressed was marked with write arrows; blue is DAPI, scale bar: 50µm. Qualification of (I) TTLL6 fluorescence intensity in SR area of CA3 region in different groups; n = 3 biological repeats. SR: stratum radiatum area: composed mostly of apical dendrites of pyramidal neurons; SP: stratum pyramidale: composed mostly of tightly arranged pyramidal cells. *P <0.05, **P < 0.01, ***P <0.001.

**
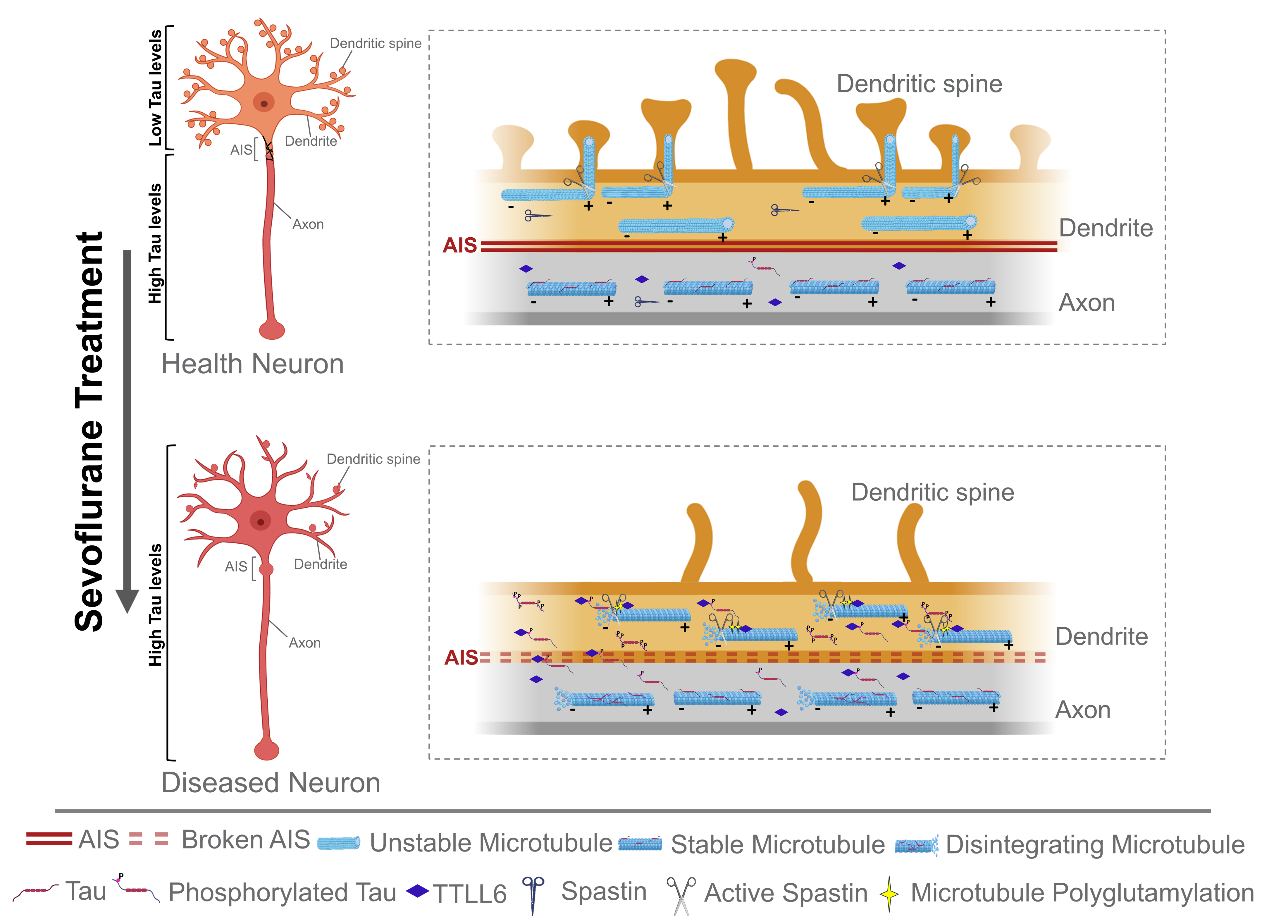
**

**Fig. S3.** **Hypothesized pathway for how Tau-TTLL6 missorting is implicated in sevoflurane-induced cognitive and synaptic plasticity deficits in neonatal mice.** Tau connects to microtubules (MT) in healthy neurons and is typically found in axons. Tau is phosphorylated and detaches from microtubules under the sevoflurane condition, causing soma and dendritic misplacement. Tau missorting induces TTLL6 mislocalization in dendrites. TTLL6 enhances α-tubulin polyglutamylation, which results in Spastin-mediated dendritic microtubule severing. The absence of microtubules hampers the process of dendritic spine invasion, causing a disruption in synaptic function and resulting in the depletion of fully developed spine.
