# Supplementary figures and images for: Repeated Exposure to Sevoflurane in Neonatal Mice Induces Cognitive and Synaptic Impairments in a TTLL6‐Mediated Tubulin Polyglutamylation Manner
Source: CNS Neurosci Ther. 2025 Apr 9;31(4):e70376. doi: 10.1111/cns.70376 (PMC11979716; doi:10.1111/cns.70376)

## Slide 1
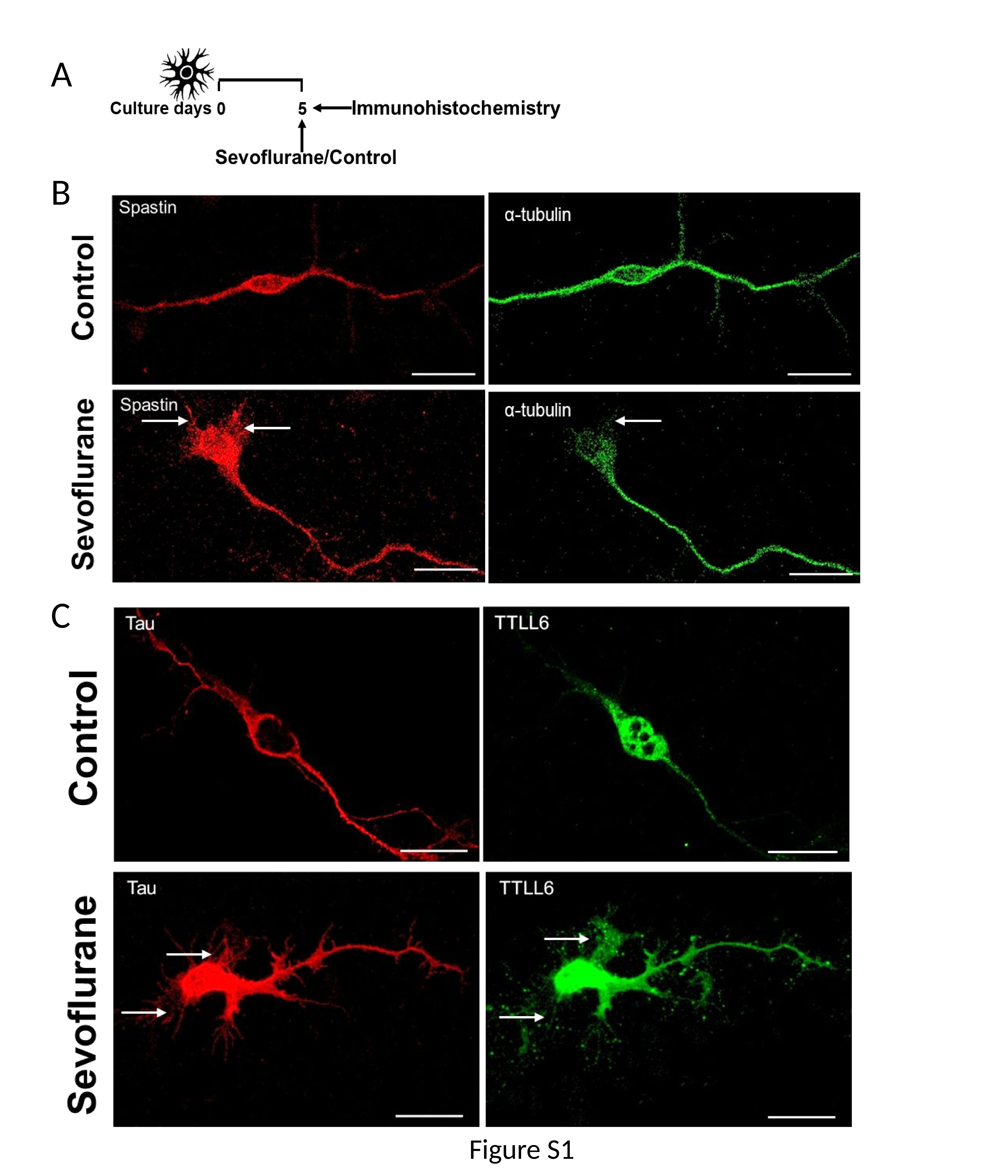

A
B
C
Figure S1

## Slide 2
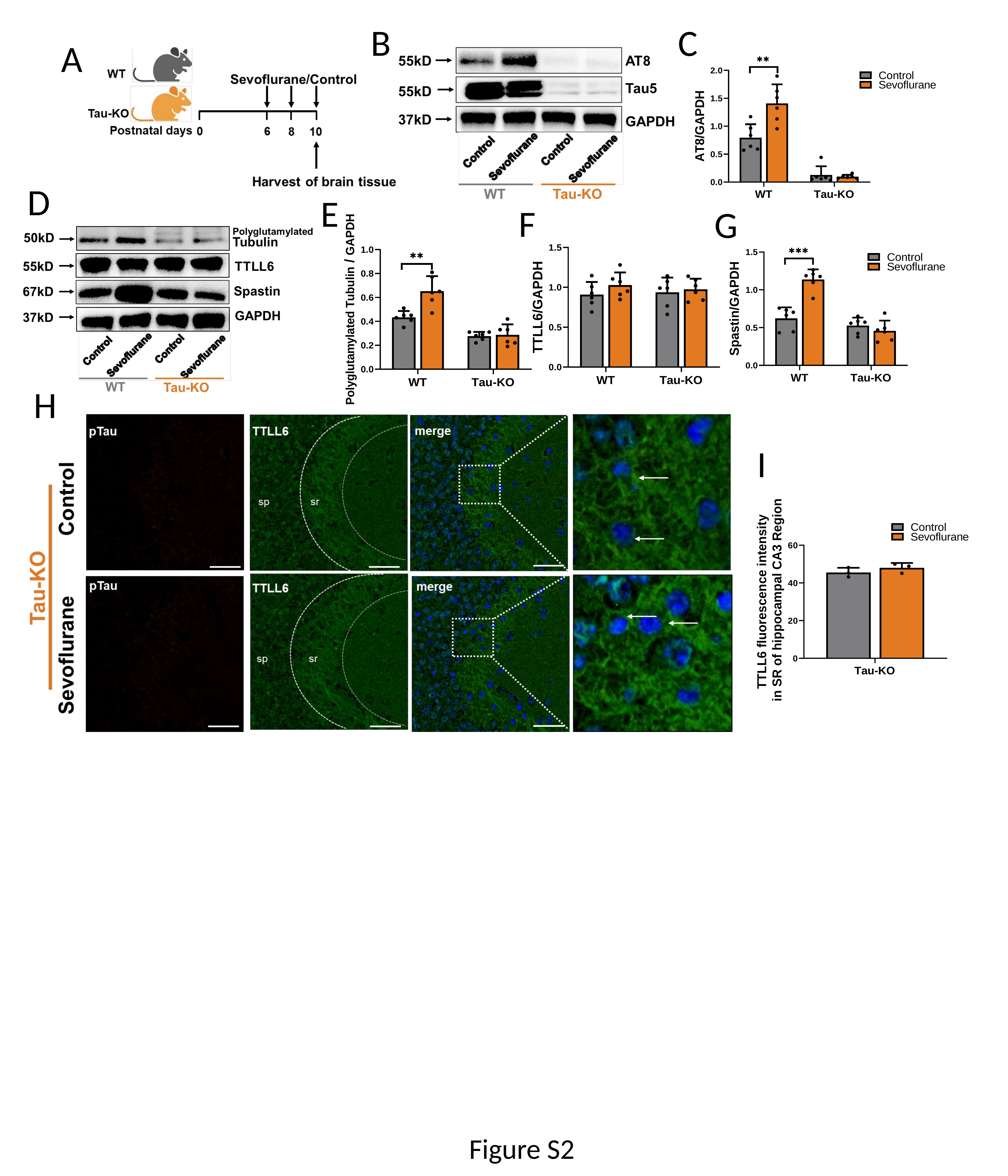

B
C
A
D
E
F
G
H
I
Figure S2

## Slide 3
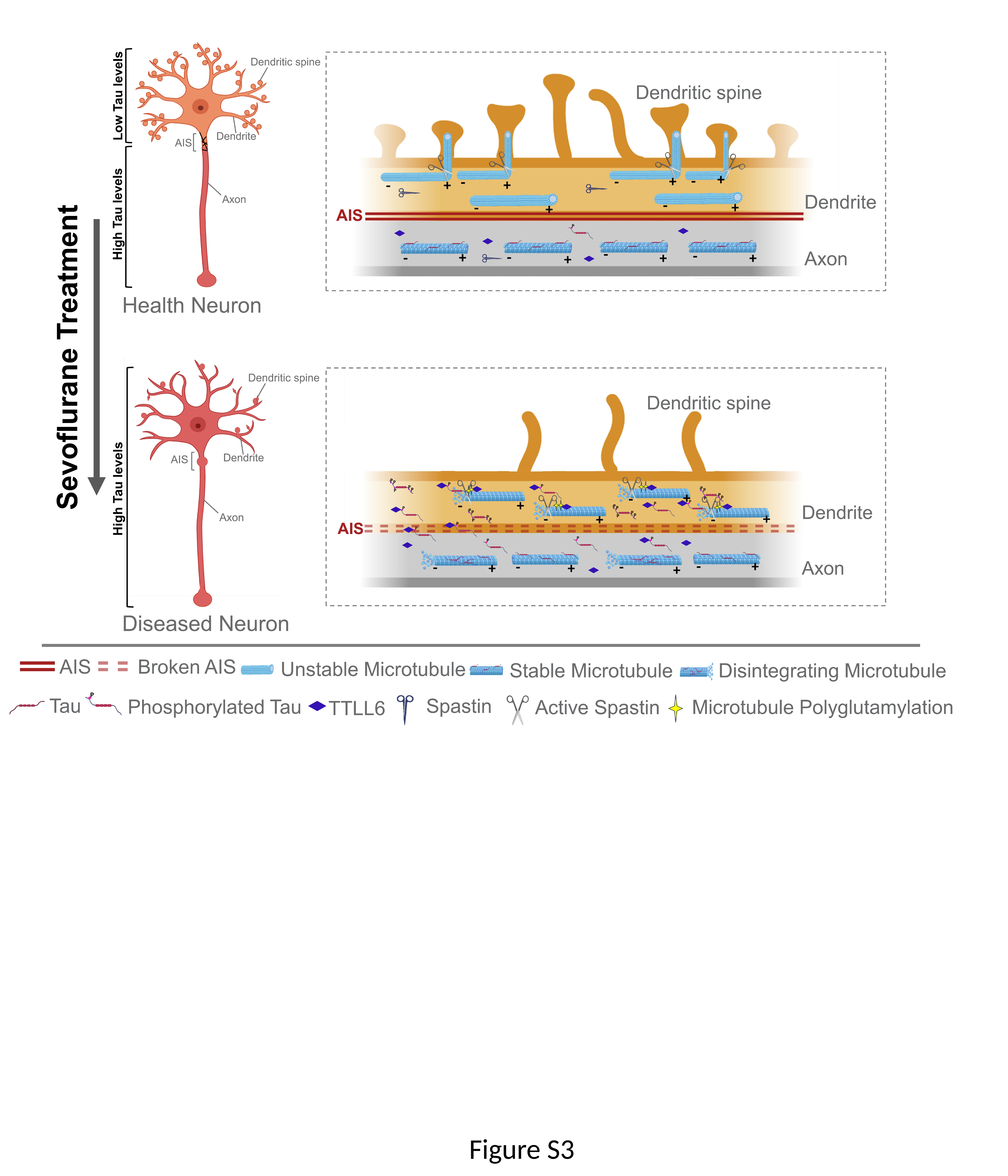

Figure S3

Supplement: Supplementary file 1 — Data S1. [file CNS-31-e70376-s001.zip › Supinfo.pptx]
